# First manifestation of cardiovascular disease according to age and sex in a Mediterranean country

**DOI:** 10.3389/fcvm.2024.1403363

**Published:** 2024-09-17

**Authors:** Emilio Ortega, Idoia Genua, Manel Mata-Cases, Mercè Roqué, Bogdan Vlacho, Jordi Real Gatius, Josep Franch-Nadal, Didac Mauricio

**Affiliations:** ^1^ Department of Endocrinology & Nutrition, Hospital Clinic Barcelona, Barcelona, Spain; ^2^ CIBER of Obesity and Nutrition (CIBEROBN), Instituto de Salud Carlos III (ISCIII), Madrid, Spain; ^3^ Instituto de Investigaciones Biomédicas August Pi I Sunyer (IDIBAPS), Barcelona, Spain; ^4^ Department of Endocrinology & Nutrition, Hospital de la Santa Creu I Sant Pau, Barcelona, Spain; ^5^ Department of Medicine, Autonomous University of Barcelona, Barcelona, Spain; ^6^ Institut d’Investigació Biomèdica Sant Pau (IIB SANT PAU), Barcelona, Spain; ^7^ DAP-Cat Group, Unitat de Suport a la Recerca Barcelona, Fundació Institut Universitari per a la Recerca a L'Atenció Primària de Salut Jordi Gol I Gurina (IDIAPJGol), Barcelona, Spain; ^8^ CIBER of Diabetes and Associated Metabolic Diseases (CIBERDEM), Instituto de Salud Carlos III (ISCIII), Madrid, Spain; ^9^ Primary Health Care Center La Mina, Gerència D'Atenció Primària Barcelona Ciutat, Institut Català de la Salut, Sant Adrià de Besòs, Spain; ^10^ Department of Cardiology, Hospital Clinic Barcelona, Barcelona, Spain; ^11^ Primary Health Care Center Raval Sud, Gerènciad’Atenció Primaria, InstitutCatalà de la Salut, Barcelona, Spain; ^12^ Department of Medicine, University of Vic—Central University of Catalonia, Vic, Spain

**Keywords:** cardiovascular events, epidemiology, risk factors, sex differences, age differences, large cohorts

## Abstract

**Background:**

Cardiovascular (CV) diseases are the most common cause of death worldwide. This study aimed to investigate the incidence and type of first CV event in a broad cohort of Spaniards, focusing on age and sex differences.

**Methods:**

This was a retrospective study using the SIDIAP database. Subjects aged 30–89 years in 2010 were included. Individuals with prevalent CV disease or atrial fibrillation were excluded. Subjects were followed until the occurrence of a CV event, death, or the study end (December 2016). CV outcomes (coronary heart disease [CHD], cerebrovascular or peripheral artery disease and heart failure [HF]) during follow-up were analyzed. Clinical, anthropometrical, and laboratory data were retrieved from clinical records.

**Results:**

Overall, 3,769,563 at-risk individuals (51.2 ± 15.2 years) were followed for a median of 7 years. The cumulative incidence of a first CV event was 6.66% (men vs. women, 7.48% vs. 5.90%), with the highest incidence (25.97%) among individuals >75 years. HF (29%) and CHD (28.8%) were the most common first events overall; in men it was CHD (33.6%), while in women it was HF and cerebrovascular disease (37.4% and 27.4%). In younger age groups, CHD was more prevalent, with HF in older age groups. Baseline CV risks factors conferred more risk in younger ages and differed between men and women.

**Conclusions:**

The incidence and type of the first CV event in this Mediterranean region were significantly influenced by age and sex. This information is relevant for tailoring primary prevention strategies including the treatment of risk factors.

## Graphical Abstract

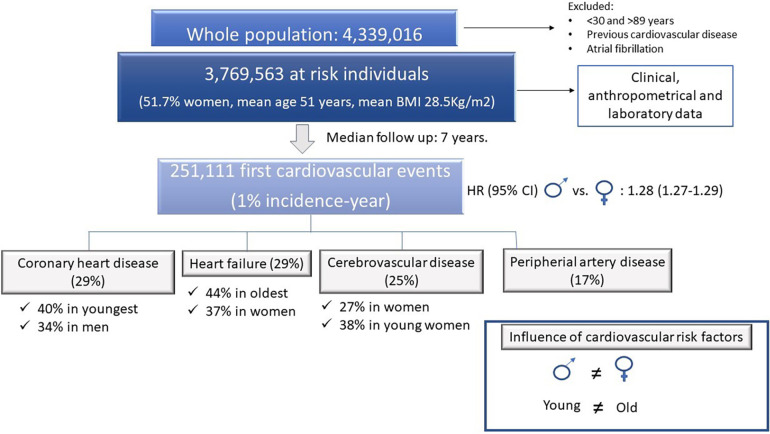

Summary of methods and main findings of the present work.

## 1 Introduction

Cardiovascular diseases (CVDs) are responsible for a high proportion of deaths in Spain and other European countries (1, 2). These diseases mainly result from atheroma plaques in the arterial walls (3). Heart failure (HF) is also a relevant manifestation of CVD and is associated with a high burden on healthcare expenditures (4, 5).

There are varied manifestations of CVD. Myocardial infarction (MI), hospitalization for unstable angina, ischemic or hemorrhagic stroke, short-distance intermittent claudication, or acute limb ischemia are probably the best-known manifestations, and the usual inclusion criteria for randomized trials evaluating preventive CVD therapies (6). However, advanced preclinical atherosclerosis (such as carotid stenosis), transient ischemic attack (TIA), revascularization procedures (not always a direct consequence of an acute event), stable initial angina or undetermined ischemic heart disease, among others, are also significant clinical manifestations of CVD. These entities also deserve clinical attention and are relevant to prevention strategies, deeper cardiovascular (CV) evaluation, hospital admission, and, importantly, changes in individuals’ quality of life or perception of health status (7).

The type and severity of CVD manifestations can vary between populations according to the country's wealth (8), genetic, cultural, and ethnic backgrounds, access to health care, and socioeconomic status. Furthermore, within populations, differences can be seen by age and sex. In addition, changes can occur over time (9). Therefore, current population-based data are needed.

Our study aimed primarily to describe the incidence of the first ever reported clinical manifestation of CVD in a cohort without previous CV events and to evaluate differences according to age groups and sex using real-world clinical data in Catalonia (Spain). As a secondary aim, we evaluated the association between baseline clinical cardiovascular risk factors and incident events, and how these associations varied by age and sex.

## 2 Materials and methods

### 2.1 Study design and data sources

The Catalan Health Institute (ICS) is the leading provider of primary healthcare services in the Catalan Health System (CatSalut), encompassing 74% of the total population. Primary care professionals from the ICS use the same computerized medical record program (e-CAP). The SIDIAP database, contains pseudo-anonymous, longitudinal, routinely collected health data from the e-CAP. The Minimum Basic Data Set of Hospital Discharges (CMBD database) and pharmacological treatments (from pharmacy-invoicing data provided by the CatSalut) are automatically linked to the SIDIAP.

Our study population consisted of individuals aged at least 30 years included in the SIDIAP dataset on January 1st, 2010 (*n* = 4,339,016). We excluded individuals with prevalent CVD (*n* = 221,888), known atrial fibrillation (*n* = 85,944), or those aged under 30 or above 89 years (*n* = 313,645). The upper (89 years) and lower (30 years) age limits were selected to represent a wide range of ages and based on previous cohorts (10, 11) and life expectancy. Furthermore, below and above these age cut-offs, CV evaluation is not systematically recommended, and prevention of a first cardiovascular event is not a clinical priority, respectively. The remaining individuals, considered at-risk or in primary prevention, were followed until the occurrence of a CV event, death, or the end of the study (December 2016). The study population was divided into five different age groups according to baseline age and sex: <35 years (young), 35–55/60 (men/women) years (early-adulthood), 55/60–65 (men/women) years (middle-adulthood), 65–75 years (young-old) and >75 years (middle-to-very old). This age grouping was based on a pragmatic, clinical, and convenient approach (12, 13). The study flowchart is summarized in Supplementary Figure S1.

This was a retrospective study using pseudo-anonymous routinely collected health data; thus, informed consent was not obtained from participants in accordance with Spanish regulations for observational studies. The Ethics Committee of the Primary Healthcare University Research Institute (IDIAP) Jordi Gol (Barcelona, Spain) approved the study on 15/03/2017: approval number P17/087.

### 2.2 Baseline characterization and outcomes

Baseline characteristics were collected as of January 1st, 2010, considered as the study index date. Variables related to age, sex, and toxic habits were collected. The alcohol consumption risk levels were defined using standard drink units (Supplementary Table S1) (1 standard drink unit equals 10 g of alcohol). The socioeconomic deprivation index was measured using the MEDEA index (14) and categorized into quintiles (Q1–Q5), where Q1 are the least deprived ones. This index uses five socioeconomic indicators: percentage of unemployment, percentage of manual workers, percentage of temporary workers, percentage of low educational level (people aged 16 years or over who are illiterate or who did not complete primary education), and percentage of low educational level in young people (aged 16–29 years). We analyzed the presence of CVD diagnoses and relevant comorbidities, according to ICD-9/ICD-10 diagnostic codes, and the use of concomitant medications for comorbidities (based on the Anatomical Therapeutic Chemical-ATC classification system). We also collected available blood pressure, anthropometric and laboratory data [lipids, creatinine, urinary albumin excretion (UAE)]. Atherogenic dyslipidemia was considered when triglycerides were >150 mg/dl and HDLc was low (<40/50 mg/dl, men/women). A diagnosis of chronic kidney disease was based on the estimated glomerular filtration rate calculated using the Chronic Kidney Disease Epidemiology Collaboration (CKD-EPI) equation and/or the urine albumin-to-creatinine ratio (UACR). Microalbuminuria was defined as a UACR of 30–299 mg/g, and macroalbuminuria as a UACR ≥ 300 mg/g. SCORE2 and SCORE2-OP for low-risk regions risk prediction algorithms were used.

Cardiovascular events were defined as the presence of ICD10/9 diagnostics codes in our database. They were classified under four major conditions, i.e., coronary heart disease (CHD), cerebrovascular disease, peripheral artery disease (PAD), and HF. CHD was defined as fatal or non-fatal MI, angina or unstable angina, undetermined ischemic heart disease, or coronary revascularization [coronary artery bypass grafting (CABG)], percutaneous coronary intervention [PCI]). Cerebrovascular disease was defined as fatal or non-fatal ischemic or hemorrhagic stroke, TIA, or intracerebral revascularization. PAD was defined as intermittent claudication, extracerebral artery stenosis, or carotid or peripheral revascularization (endovascular, stenting, or surgical bypass). HF included congestive/acute HF and other HF diagnoses (systolic and diastolic, chronic, or undetermined). Specific diagnosis codes for CVD can be found on Supplementary Table S2. The date of people's death during the study period was also retrieved from the SIDIAP database.

### 2.3 Statistical analysis

We report baseline characteristics using frequencies and percentages for categorical variables and mean and standard deviation (SD) or median and quartiles for continuous variables. CV manifestations’ cumulative incidence and incidence rates were directly calculated without assuming any underlying probability distribution. Therefore, the cumulative incidence was calculated as the ratio of the number of incident cases to the population at risk. The incidence density, or incidence rate, was calculated to quantify the occurrence of new cases within the study population over the follow-up period. For each event of interest, the overall incidence rate (IR) and age-sex-specific incidence were computed as the number of incident events divided by the person-years (PYs) during the follow-up and were expressed per 100 PYs.

Hazard ratios (HR) and 95% confidence intervals (95% CI) were computed for each baseline characteristic. A complete-case analysis was performed, including only subjects with available data for each respective variable. Since missing data were not imputed, cases with incomplete information were excluded from the analysis. Crude HRs in Supplementary Table S5 were computed using the “compareGroups” R package (Version 4.6.0) (15), using Cox proportional hazard regression models. For the age-adjusted HRs, we used the coxph function from the {survival} R package, which fits a Cox proportional hazards regression model based on the counting process formulation of Andersen and Gill (16, 17). All statistical analyses were performed using the free R statistical software version 3.6.1 (https://www.r-project.org/).

## 3 Results

### 3.1 Baseline characteristics

A total of 3,769,563 individuals were included in this primary prevention cohort. At baseline, the mean age was 51.2 (SD = 15.2) years. Most individuals were in the early-adulthood group (52.7%), followed by the young-group (14.9%), the middle-adulthood group (11.7%), the young-old group (11.1%), and the middle-to-very old group (9.6%) (*p* < 0.001) (Supplementary Table S3). There were more women (51.7%) than men, especially in the middle-to-very old group (63.9%). The average BMI was 28.5 ± 5.07, 6.6% had type 2 diabetes (T2DM), 34.2% obesity, 19.5% hypertension, 12.1% received statin treatment and 19.6% were current smokers (Supplementary Table S3).

### 3.2 Incidence rate, frequency and type of cardiovascular manifestation

**Figure 1:**
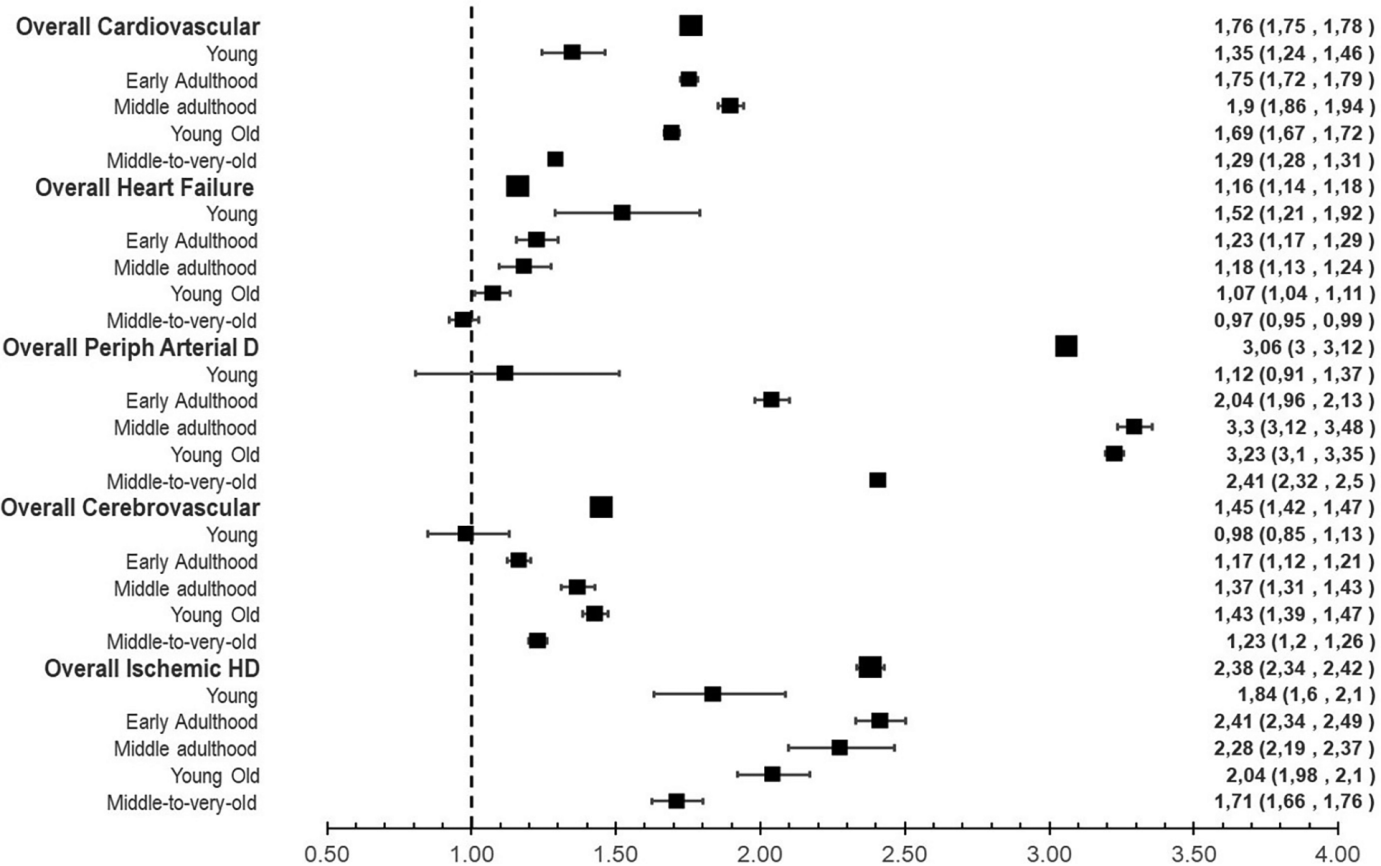
Risk ratio of cardiovascular disease manifestation by age group and type of manifestation: men vs. women. Data indicate hazard ratios and 95% confidence intervals for the risk of first cardiovascular event for men (women as the reference category). Age-adjusted (overall categories) and crude (age group categories) HR and 95% CI are shown. Ischemic HD, ischemic heart disease; Periph Arterial D, peripheral arterial disease.

Between January 2010 and December 2016 (median follow-up of seven years), 251,111 (6.66%) from a total of 3,769,563 (25,058,095 PYs) at-risk individuals had a CV event (Table 1). The cumulative incidence of CVD was higher in men than in women (7.5% vs. 5.9%; *p* < 0.001) and increased with age, reaching 24.3% and 28.9% in the oldest age groups for women and men, respectively (Table 1). Compared with women, men had a 24% higher risk of having a first CV event [age-adjusted HR (95% CI), 1.76 (1.75–1.78)], with the middle-adulthood age group having the highest excess risk (Figure 1). This higher age-adjusted risk was more evident in the case of peripheral arterial [3.06 (3.00–3.12)] and coronary heart disease [2.38 (2.34–2.42)] and less manifest for stroke [1.45 (1.42–1.47)], and, particularly, heart failure [1.16 (1.14–1.18)] (Figure 1). The latter case was mainly explained by the higher risk (and large proportion, Table 2) of heart failure events observed in women in the middle-to-very-old age group.

**Table 1:** Incidence of the first cardiovascular manifestation.

	Population (*n*)	Events (*n*)	Person-time (years)	Incidence rate (% persons-year)	Cumulative incidence (%)
All	3,769,563	251,111	25,058,095	1.002	6.662
Women	1,949,847	115,055	13,042,406	0.882	5.901
Men	1,819,716	136,056	12,015,688	1.132	7.477
Age and sex groups					
Y (Young <35 years)	561,847	2,361	3,920,581	0.06	0.42
Y-women	271,639	968	1,896,802.1	0.051	0.356
Y-men	290,208	1,393	2,023,779	0.069	0.48
EA (Early adulthood 35–55/60 years, M/F)	1,986,287	48,722	13,679,093	0.356	2.453
EA-women	1,053,464	19,198	7,283,955.3	0.264	1.822
EA-men	932,823	29,524	6,395,137.7	0.462	3.165
MA (Middle adulthood 55/60–65 years, M/F)	439,931	41,974	2,884,920	1.455	9.541
MA-women	157,601	9,853	1,059,917.6	0.93	6.252
MA-men	282,330	32,121	1,825,002.8	1.76	11.377
YO (Young old, 65–75 years)	418,105	63,666	2,619,724	2.43	15.227
YO-women	234,879	28,565	1,516,158.8	1.884	12.162
YO-men	183,226	35,101	1,103,565.7	3.181	19.157
MVO (Middle-to-very-old, >75 years)	363,393	94,388	1,953,776	4.831	25.974
MVO-women	232,264	56,471	1,285,572.6	4.393	24.313
MVO-men	131,129	37,917	668,203.1	5.674	28.916

Y, young, <35 years; EA, early adulthood, 35–55/60 years (men/women); MA, middle adulthood, 55/60–65 (men/women); YO, young old, 65–75 years; and MVO, middle-to-very old, >75 years. Overall *p* value when compared men vs. women <0.001; *p* value through age groups <0.001.

**Table 2:** First territory affected by cardiovascular disease in the entire population and in women and men separately.

CV manifestation	Sex	[ALL]	Y (<35 years)	EA (35–55/60 years)	MA (55/60–65 years)	YO (65–75 years)	MVO (>75 years)
Number of events	Entire population	*N* = 251,111	*N* = 2,361	*N* = 48,722	*N* = 41,974	*N* = 63,666	*N* = 94,388
	Women	*N* = 115,055	*N* = 968	*N* = 19,198	*N* = 9,853	*N* = 28,565	*N* = 56,471
	Men	*N* = 136,056	*N* = 1,393	*N* = 29,524	*N* = 32,121	*N* = 35,101	*N* = 37,917
Heart failure	Entire population	72,856 (29.0%)	307 (13.0%)	6,465 (13.3%)	6,988 (16.6%)	17,202 (27.0%)	41,894 (44.4%)
	Women	43,079 (37.4%)	117 (12.1%)	3,115 (16.2%)	2,306 (23.4%)	9,685 (33.9%)	27,856 (49.3%)
	Men	29,777 (21.9%)	190 (13.6%)	3,350 (11.3%)	4,682 (14.6%)	7,517 (21.4%)	14,038 (37.0%)
Peripheral artery disease	Entire population	43,564 (17.3%)	375 (15.9%)	9,559 (19.6%)	9,652 (23.0%)	12,227 (19.2%)	11,751 (12.4%)
	Women	13,914 (12.1%)	171 (17.7%)	3,428 (17.9%)	1,449 (14.7%)	3,658 (12.8%)	5,208 (9.22%)
	Men	29,650 (21.8%)	204 (14.6%)	6,131 (20.8%)	8,203 (25.5%)	8,569 (24.4%)	6,543 (17.3%)
Cerebrovascular disease	Entire population	62,489 (24.9%)	747 (31.6%)	12,458 (25.6%)	9,907 (23.6%)	16,308 (25.6%)	23,069 (24.4%)
	Women	31,577 (27.4%)	365 (37.7%)	6,163 (32.1%)	2,958 (30.0%)	8,014 (28.1%)	14,077 (24.9%)
	Men	30,912 (22.7%)	382 (27.4%)	6,295 (21.3%)	6,949 (21.6%)	8,294 (23.6%)	8,992 (23.7%)
Coronary heart disease	Entire population	72,202 (28.8%)	932 (39.5%)	20,240 (41.5%)	15,427 (36.8%)	17,929 (28.2%)	17,674 (18.7%)
	Women	26,485 (23.0%)	315 (32.5%)	6,492 (33.8%)	3,140 (31.9%)	7,208 (25.2%)	9,330 (16.5%)
	Men	45,717 (33.6%)	617 (44.3%)	13,748 (46.6%)	12,287 (38.3%)	10,721 (30.5%)	8,344 (22.0%)

Data indicate number and percentage. Y, young, <35 years; EA, early adulthood, 35–55/60 y (men/women); MA, middle adulthood, 55/60–65 (men/women); YO, young old, 65–75 years; and MVO, middle-to-very old, >75 years. *p* < 0.001 through age groups for the entire population, men and women.

**Figure 2:**
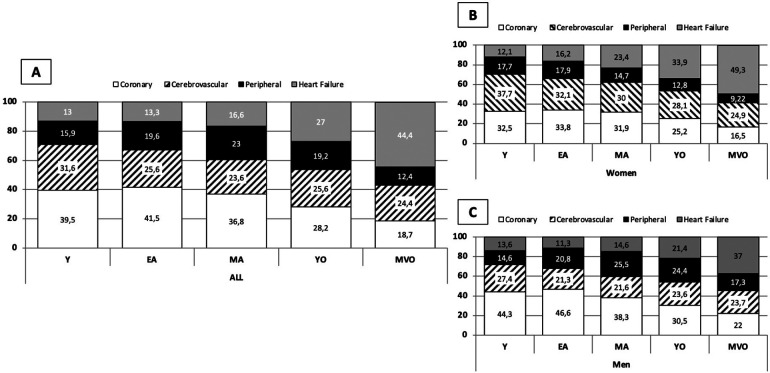
First manifestation of cardiovascular disease in the entire population and in women and men separately. **(A)** entire population; **(B)** women; **(C)**, men. EA, early adulthood, 35-55/60 y (men/women); MA, middle adulthood, 55/60–65 years (men/women); MVO, middle-to-very old, >75 years; Y, young, <35 years and YO, young old, 65–75 years.

Figure 2A; Table 2 show the distribution and absolute numbers of the first recorded CVD manifestation by age group. Overall, HF (29%) and CHD (28.8%) were the most frequent manifestations. CHD accounted for 39.5% and 41.5% of total first events in young and early-adulthood individuals and decreased thereafter. The opposite trend was observed for HF, ranging from 44.4% of all incident events in the oldest to 13% in the youngest group. Cerebrovascular disease followed a similar, although less evident, pattern to that observed with CHD. Finally, the proportion of PAD events was slightly lower in the youngest and oldest groups, but no marked age group differences existed.

Some differences in the distribution of the first CVD manifestation by age in men and women were apparent (Figures 2B,C; Table 2). CHD was the most prevalent manifestation in men (33.6%), and HF and cerebrovascular disease in women (37.4% and 27.4%, respectively). In women, there was a sharp age-related increase in the proportion that had HF as a first manifestation, from 12.1% in the young group to 49.3% in the middle-to-very old group. The proportion of women with PAD as a first manifestation decreased from 17.7% in the young group to 9.2% in the middle-to-very old group, while the proportion of men with PAD increased until the age of 75 years (young to young-old groups, 14.6%–24.4%, respectively). The proportion of cerebrovascular events varied less in men (from 21.3% to 27% across age groups) than in women, in whom a progressive decrease from 37.7% in the young group to 24.9% in the middle-to-very old group was observed. The proportion with CHD by age group for men and women followed a similar trend to that described for the total population.

### 3.3 Cardiovascular disease subtypes

HF, CHD, PAD, and cerebrovascular disease represent umbrella terms for more specific CVD manifestations; these specific conditions followed a similar sex- and age-pattern as their umbrella category described above (Supplementary Table S4; Supplementary Figure S2). Congestive/acute HF represented approximately 40% of the new HF diagnoses. Ischemic stroke and TIA were the most prevalent manifestations of cerebrovascular disease, with TIA being more frequent in women. The proportion with hemorrhagic stroke as a first manifestation of CVD was higher in the youngest age group (6.18%). Myocardial infarction (MI) was the most prevalent manifestation in young and early adulthood (17.7% and 19.6%, respectively).

### 3.4 Baseline characteristics and risk of a first cardiovascular event

**Figure 3:**
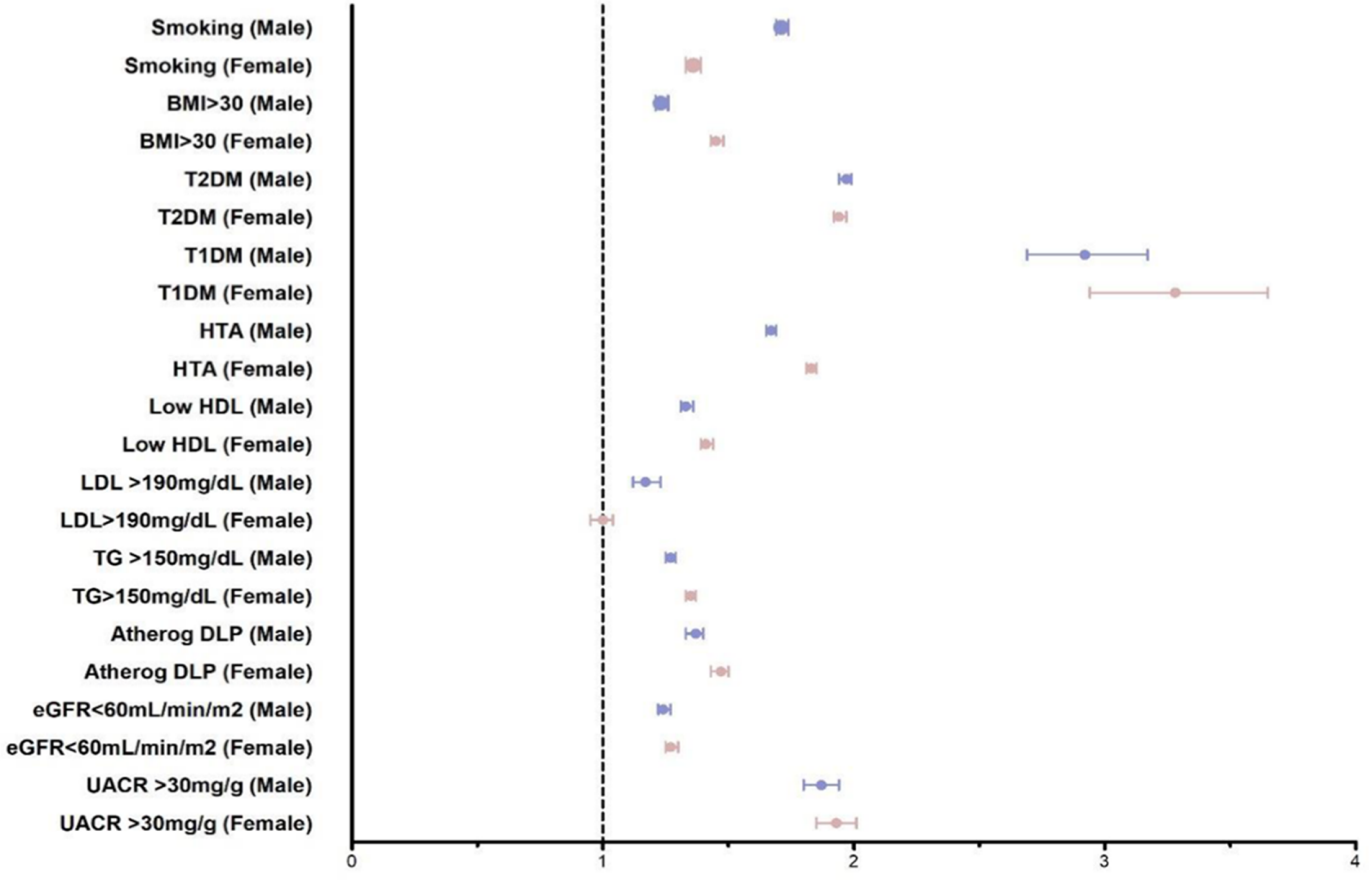
Age-adjusted risk of having a first cardiovascular event according to baseline characteristics and sex. Data expressed as Hazard Ratio (event vs. no event) and 95%CI. Atherog DLP, atherogenic dyslipidemia; BMI, body mass index (Kg/m^2^); eGFR, estimated glomerular filtration rate; HDL, high-density lipoprotein; HTA, hypertension; LDL, low-density lipoprotein; T1DM, type 1 diabetes; T2DM, type 2 diabetes, TG, tryglicerides; UACR, urinary albumin-to-creatinin ratio.

Baseline characteristics of subjects with and without incident CV events and hazard ratios by age group and sex are shown in Figure 3; Supplementary Table S5. Overall, the presence of risk factors in younger age groups compared with older age groups were associated with a significant excess risk of a first CV event. Although moderate and high CV risk (by SCORE2 or SCORE2-OP) were more frequent in men, they conferred similar risks in men and women, particularly in the early-adulthood and middle-adulthood groups (Supplementary Table S5).

Smoking was more prevalent in men and conferred a higher risk of CVD for all age groups. Several metabolic conditions (low HDLc, high BMI, hypertension, and non-alcoholic fatty liver) were more prevalent in women and were associated with a higher risk of CVD in women than men in most age groups. Atherogenic dyslipidemia was also more prevalent in women (except for the young and early-adulthood groups) and was associated with a higher excess risk of CVD. T2DM was more prevalent in men, but women with T2DM had a higher risk of CVD than men with T2DM when compared with their non-diabetic counterparts for all ages, except for young men vs. young women: HR [95% CI] 7.48 [4.96;11.3] vs. 4.21 [2.10;8.44], respectively (Supplementary Table S5). Type 1 diabetes (T1DM) was associated with a higher risk of CVD in women. Socioeconomic deprivation prevalence (U5, highest quintile) was slightly more prevalent in men, but the HR for CVD risk was higher for women.

### 3.5 Mortality

From the initial 3,769,563 individuals, 188,263 (4.99%) (4.59% women; 5.43% men) died during follow-up (Supplementary Table S6). Before the date of death, 30.2% of these individuals (a similar percentage in men and women) had a CV condition (Supplementary Table S7).

## 4 Discussion

In this study, we report the incidence of CVD and describe the first reported manifestation in adults without previous CVD living in a low-risk Mediterranean country. We found an overall 1% person-year incidence rate of a first CV event during a median seven-year follow-up. There are considerable differences in CV incidence worldwide (18) and across Europe (1). Spain is a low-risk country with one of the lowest incidences of CVD and CV deaths in Europe (19). In 2017, Europe's median incidence rate was estimated to be 1,133 per 100,000 inhabitants, while in Spain it was 1,091/100,000 inhabitants (9); the latter is in line with our results. In the FRESCO Study, performed in Spain between 1992 and 2005 with 11 population cohorts, a similarly low incidence of CV events was found (20).

CVD incidence varies with age (21), the most crucial driver of CV disease (22). In our study, the incidence of CV events increased from 0.06% person-year in younger ages to 4.83% in the oldest age group. In a US study evaluating risk factors in 4,883 individuals (>65 years) at primary prevention, the incidence of CV events ranged from 1.7% to 5.54% person-year (21). However, we considered a wider age range and a different age grouping approach. Our strategy was based on usual clinical practice in our system, age range at which systematic CV risk evaluation is recommended (13, 22), age for considering premature CVD and a common cut-off (75 years) for “elderly” description in Spain. The clinical recommendation is to individualize CV prevention in young (<40–45 years) and older (>75 years) individuals since these individuals are usually underrepresented in clinical trials (23, 24).

HF (29%) and CHD (28.8%) were our population's most common first CV manifestations. Ischemic heart disease has been previously identified as the most frequent type of CV event (9) and cause of CV death (25). However, HF, which usually remains underdiagnosed up to several years before the onset of classical clinical manifestations or hospital admission (4), accounted for a large part of CV events. This high HF incidence was mainly accounted for in the oldest age group, a finding that is in agreement with previous studies (4, 26, 27) and probably due to some known risk factors such as hypertension, diabetes, obesity (28) or prior asymptomatic cardiac disease.

Previous studies show that absolute CV risk is higher in men than women; however, this risk changes by age and type of CV disease manifestation (29, 30). Sex differences are less significant at older ages and for events such as cerebrovascular disease and HF (29, 31). In our study, the most common first manifestations of CVD were HF and cerebrovascular disease in older individuals and CHD in younger individuals. In women, cerebrovascular disease (especially at younger ages) and HF (in older ages) were more frequent, whereas in men, CHD prevailed. In the Netherlands, a country with a higher CV risk than ours, the incidence rate of the first CV event in individuals ≥55 years was 3.55% person-year. Women and participants >75 years had more frequent HF and cerebrovascular disease (32). Similar results were found in an extensive prospective study of 1,937,360 individuals in England with a median follow-up of 6 years and a total of 114,859 CV events (31). In this study, stroke and HF were the most common first CVD manifestations in older adults (>80 years) and women. Non-fatal MI was the most frequent in men.

Roughly, 5% of the individuals died during the follow-up, and approximately 30% of these individuals suffered a CV event before the date of death. Unfortunately, the specific cause of death was not available in this study. Although direct CV mortality (for example, sudden cardiac arrest or fatal massive stroke) may not be a prevalent first CVD manifestation (13, 20, 33), consequences of a previous CV event may have influenced this disproportionate high mortality rate observed in individuals who suffered a CV event.

We also described the prevalence of several traditional and non-traditional risk factors and their association with incident events. Of note, women who had metabolic risk factors appeared to have higher risk of a first incident CV event than men with the same risk factors. This was particularly evident in the two youngest age groups. This was also true for T1DM and T2DM, with higher excess risk for a CV event in women (34). In people with T1DM, the risk was highest in young women, while in T2DM the risk was highest in the youngest men, with a similar risk for men and women in older groups. Diabetes confers a stronger excess risk of cardiovascular events in women with diabetes as compared with men. Women with diabetes partially lose their usual protection against CVD (35). The excess CV risk in women with other established CV risk factors has also been previously described in the literature (36). However, sex-specific mechanisms are not fully understood (36, 37). In women, hypertension, which also confers higher excess CV risk as compared with men, is the main contributor to cardiovascular mortality (36). Moreover, in T2DM, BMI had the most significant contribution to heart failure risk, the most prevalent first manifestation of CVD in women in our study (38). Smoking, however, was more prevalent and associated with a higher CV risk in men. Indeed, smoking in men has been reported to contribute to the sex differences seen with coronary and peripheral diseases (39). These findings, previously described in the literature (36), add information and relevance to sex differences in CVD risk.

Our data may help inform current prevention strategies in our Mediterranean country. These sex and age differences should prompt attention to a better evaluation and treatment, mainly in terms of intensity and goal setting, of risk factors in primary prevention strategies that could be partially redirected from a mainly lipid-centric, a leading contributor to CHD (40), to a more adipo/diabetes-centric and blood pressure approach, relevant determinants of HF and stroke. This, however, should be addressed in dedicated clinical trials. Also, despite CVD incidence being lower in young people, when risk factors are already present at this age, more intense and proactive management should be considered in prevention programs (41). Finally, HF and PAD (leading manifestations in our study) are seldom prioritized when assessing CV risk. Predictive equations based on data not incorporating cerebrovascular disease (42) and/or non-fatal HF (43) outcomes may underestimate the risk, particularly in women and the elderly.

The major strengths are the population cohort used for the data analysis in a real-world scenario. The main limitations are inherent to the study's retrospective nature and potential differences in the clinical judgment of primary physicians when reporting diagnoses. However, SIDIAP is a well-validated dataset that has been extensively used for healthcare research (44–47). Further, the significant proportion of missing data could have influenced the baseline characteristics’ cardiovascular risk estimation (Supplementary Table S5). The statistical analyses were done using a complete-case analysis approach, using only the individuals with complete information in each analysis. However, this was a secondary exploratory aim in this study, and more importantly, missing data did not affect the main aim of the study aim, i.e., the incidence of cardiovascular events. On the other hand, a high proportion of individuals without information on variables associated with CV health (not screened or not proactively studied) might indicate a healthier population. In this scenario, the incidence rate would have been underestimated, although, the incidence rate of CV events was consistent with other reports in similar populations. An apparent low risk in older ages can be explained by the fact that patients with previous CV events have been excluded. Finally, our data are only informative for individuals at primary prevention; therefore, our findings are not applicable for those subjects with a previous event who were excluded from this study.

In conclusions, our data show a low incidence of CV events in a Mediterranean country with significant differences in the first manifestation of cardiovascular disease according to sex and age. These data are informative to clinicians and health authorities to help in planning and implementing prevention strategies in different age groups and sexes, and in the design of clinical trials to continue decreasing cardiovascular events, particularly at younger ages and women.

## Data Availability

The data analyzed in this study is subject to the following licenses/restrictions: the data analyzed in this study is subject to the following licenses/restrictions: The data controller for SIDIAP does not allow the sharing of raw data. The datasets analyzed for this study can be found in the following link: https://github.com/jrealgatius/PRECAV/blob/master/codi_emilio/2_preparacio_emilio.Rmd. Requests to access these datasets should be directed to Emilio Ortega, eortega1@clinic.cat.
