# Supplementary material for: First manifestation of cardiovascular disease according to age and sex in a Mediterranean country
Source: Front Cardiovasc Med. 2024 Sep 17;11:1403363. doi: 10.3389/fcvm.2024.1403363 (PMC11443696; doi:10.3389/fcvm.2024.1403363)
Supplement: Supplementary file 1 [file Datasheet1.pdf]

Figure S1. Study flowchart

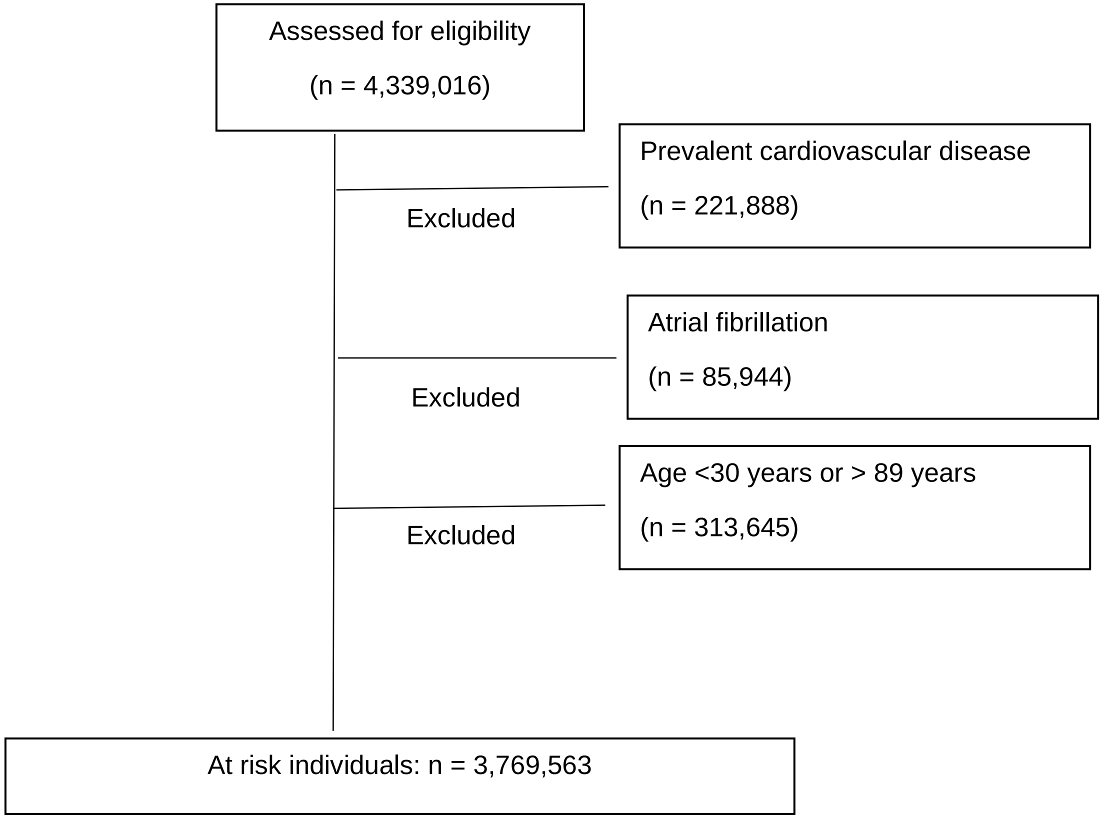

Table S1: Categories for alcohol risk consumption.

Classification of the risk	Male	Female
Risc 0 No Drinker	No alcohol consumption	No alcohol consumption
Risc 1 Low risk of alcohol consumption	Consumption of <20 SDU/weekly	Consumption of <17 SDU/weekly
Risc 2 High risk of alcohol consumption	Consumption of <28 SDU/weekly or:<br>• Having <16 years or<br>• Manages highly dangerous machines or<br>• Takes any drugs that interact with the alcohol	Consumption of <17 SDU/weekly or:<br>• Being pregnant<br>• Having <16 years or<br>• Manages highly dangerous machines or<br>• Takes any drugs that interact with the alcohol
	Consumption ≥ 28SDU/weekly	Consumption ≥ 17 SDU/weekly
	Sporadic con consumption of ≥ 6 SDU in a short period or at least once a month.	Sporadic con consumption of ≥ 5 SDU in a short period or at least once a month.

SDU: standard drink units (1 SDU = 10g alcohol)

Table S2. International Classification of Diseases and Related Health Problems codes for cardiovascular disease used in this report

ICD9/10	CODE	Description (native Catalan language)	TERRITORY	MANIFESTATION
CIM10	G45	ATACS D'ISQUÈMIA CEREBRAL TRANSITÒRIA I SÍNDROMES AFINS	CEREBROVASCULAR	TRANSIENT ISCHEMIC ATTACK (TIA)
CIM10	G45.0	SÍNDROME ARTERIAL VERTEBROBASILAR	CEREBROVASCULAR	TRANSIENT ISCHEMIC ATTACK (TIA)
CIM10	G45.1	SÍNDROME DE L'ARTÈRIA CARÒTIDA (HEMISFÈRIC)	CEREBROVASCULAR	TRANSIENT ISCHEMIC ATTACK (TIA)
CIM10	G45.2	SÍNDROMES ARTERIALS PRECEREBRALS BILATERALS I MÚLTIPLES	CEREBROVASCULAR	TRANSIENT ISCHEMIC ATTACK (TIA)
CIM10	G45.3	AMAUROSI FUGAÇ	CEREBROVASCULAR	TRANSIENT ISCHEMIC ATTACK (TIA)
CIM10	G45.4	AMNÈSIA GLOBAL TRANSITÒRIA	CEREBROVASCULAR	TRANSIENT ISCHEMIC ATTACK (TIA)
CIM10	G45.8	ALTRES ISQUÈMIES CEREBRALS TRANSITÒRIES I SÍNDROMES AFINS	CEREBROVASCULAR	TRANSIENT ISCHEMIC ATTACK (TIA)
CIM10	G45.9	ISQUÈMIA CEREBRAL TRANSITÒRIA, SENSE ALTRA ESPECIFICACIÓ	CEREBROVASCULAR	TRANSIENT ISCHEMIC ATTACK (TIA)
CIM10	G46	SÍNDROMES VASCULARS ENCEFÀLIQUES EN MALALTIES CEREBROVASCULARS (I60- 167+)	CEREBROVASCULAR	ISCHEMIC STROKE
CIM10	G46.0	SÍNDROME DE L'ARTÈRIA CEREBRAL MITJANA (I66.0+)	CEREBROVASCULAR	ISCHEMIC STROKE
CIM10	G46.1	SÍNDROME DE L'ARTÈRIA CEREBRAL ANTERIOR (I66.1+)	CEREBROVASCULAR	ISCHEMIC STROKE
CIM10	G46.2	SÍNDROME DE L'ARTÈRIA CEREBRAL POSTERIOR (I66.2+)	CEREBROVASCULAR	ISCHEMIC STROKE
CIM10	G46.3	SÍNDROMES APOPLÈTIQUES DE LA TIJA ENCEFÀLICA (I60-I67+)	CEREBROVASCULAR	ISCHEMIC STROKE
CIM10	G46.4	SÍNDROME D'INFART CEREBEL·LÓS (I60-I67+)	CEREBROVASCULAR	ISCHEMIC STROKE
CIM10	G46.5	SÍNDROME LACUNAR MOTOR PURA (I60-I67+)	CEREBROVASCULAR	ISCHEMIC STROKE
CIM10	G46.6	SÍNDROME LACUNAR SENSORIAL PURA (I60-I67+)	CEREBROVASCULAR	ISCHEMIC STROKE
CIM10	G46.7	ALTRES SÍNDROMES LACUNARS (I60-I67+)	CEREBROVASCULAR	ISCHEMIC STROKE
CIM10	G46.8	ALTRES SÍNDROMES VASCULARS ENCEFÀLICS EN MALALTIES CEREBROVASCULARS (I160-167+)	CEREBROVASCULAR	ISCHEMIC STROKE
CIM10	I11.0	MALALTIA CARDÍACA HIPERTENSIVA AMB INSUFICIÈNCIA CARDÍACA (CONGESTIVA)	HEART FAILURE	HEART FAILURE_CONGESTIVE
CIM10	I13.0	MALALTIA CARDIORENAL HIPERTENSIVA AMB INSUFICIÈNCIA CARDÍACA (CONGESTIVA)	HEART FAILURE	HEART FAILURE_CONGESTIVE
CIM10	I13.2	MALALTIA CARDIORENAL HIPERTENSIVA AMB INSUFICIÈNCIA CARDÍACA (CONGESTIVA) I INSUFICIÈNCIA RENAL	HEART FAILURE	HEART FAILURE_CONGESTIVE
CIM10	I20	ANGINA DE PIT	CORONARY	ANGOR
CIM10	I20.0	ANGINA INESTABLE	CORONARY	ANGOR
CIM10	I20.1	ANGINA DE PIT AMB ESPASME DOCUMENTAT	CORONARY	ANGOR
CIM10	I20.8	ALTRES FORMES ESPECÍFIQUES D'ANGINA DE PIT	CORONARY	ANGOR
CIM10	I20.9	ANGINA DE PIT, NO ESPECIFICADA	CORONARY	ANGOR
CIM10	I21	INFART AGUT DE MIOCARDI	CORONARY	MIOCARDIAL INFARCTION

CIM10	I21.0	INFART TRANSMURAL AGUT DE MIOCARDI DE LA PARET ANTERIOR	CORONARY	MIOCARDIAL INFARCTION
CIM10	I21.1	INFART TRANSMURAL AGUT DE MIOCARDI DE LA PARET INFERIOR	CORONARY	MIOCARDIAL INFARCTION
CIM10	I21.2	INFART TRANSMURAL AGUT DE MIOCARDI D'ALTRES LLOCS	CORONARY	MIOCARDIAL INFARCTION
CIM10	I21.3	INFART TRANSMURAL AGUT DE MIOCARDI, DE LOCALITZACIÓ NO ESPECIFICADA	CORONARY	MIOCARDIAL INFARCTION
CIM10	I21.4	INFART SUBENDOCÀRDIC AGUT DE MIOCARDI	CORONARY	MIOCARDIAL INFARCTION
CIM10	I21.9	INFART AGUT DE MIOCARDI, SENSE ALTRA ESPECIFICACIÓ	CORONARY	MIOCARDIAL INFARCTION
CIM10	I22	INFART SUBSEGÜENT DEL MIOCARDI	CORONARY	MIOCARDIAL INFARCTION
CIM10	I22.0	INFART SUBSEGÜENT DE MIOCARDI DE LA PARET ANTERIOR	CORONARY	MIOCARDIAL INFARCTION
CIM10	I22.1	INFART SUBSEGÜENT DE MIOCARDI DE LA PARET INFERIOR	CORONARY	MIOCARDIAL INFARCTION
CIM10	I22.8	INFART SUBSEGÜENT DE MIOCARDI D'ALTRES LLOCS	CORONARY	MIOCARDIAL INFARCTION
CIM10	I22.9	INFART SUBSEGÜENT DE MIOCARDI, DE PART NO ESPECIFICADA	CORONARY	MIOCARDIAL INFARCTION
CIM10	I23	ALGUNES COMPLICACIONS POSTERIORS A L'INFART AGUT DE MIOCARDI	CORONARY	MIOCARDIAL INFARCTION
CIM10	I23.0	HEMOPERICARDI COM A COMPLICACIÓ PRESENT I POSTERIOR A L'INFART AGUT DE MIOCARDI	CORONARY	MIOCARDIAL INFARCTION
CIM10	I23.1	DEFECTE DE L'ENVÀ AURICULAR COM A COMPLICACIÓ PRESENT I POSTERIOR A L'INFART DE MIOCARDI	CORONARY	MIOCARDIAL INFARCTION
CIM10	I23.2	DEFECTE DE L'ENVÀ VENTRICULAR COM A COMPLICACIÓ PRESENT I POSTERIOR A L'INFART DE MIOCARDI	CORONARY	MIOCARDIAL INFARCTION
CIM10	I23.3	RUPTURA DE LA PARET CARDÍACA SENSE HEMOPERICARDI, COM A COMPLICACIÓ PRESENT I POSTERIOR A L'INFART AGUT DE MIOCARDI	CORONARY	MIOCARDIAL INFARCTION
CIM10	I23.4	RUPTURA DE CORDES TENDINOSES COM A COMPLICACIÓ PRESENT I POSTERIOR A L'INFART AGUT DE MIOCARDI	CORONARY	MIOCARDIAL INFARCTION
CIM10	I23.5	RUPTURA DE MÚSCUL PAPIL·LAR COM A COMPLICACIÓ PRESENT I POSTERIOR A L'INFART AGUT DE MIOCARDI	CORONARY	MIOCARDIAL INFARCTION
CIM10	I23.6	TROMBOSI AURICULAR (APÈNDIX AURICULAR) I VENTRICULAR COM A COMPLICACIÓ PRESENT I POSTERIOR A L'INFART AGUT DE MIOCARDI	CORONARY	MIOCARDIAL INFARCTION
CIM10	I23.8	ALTRES COMPLICACIONS POSTERIORS A L'INFART AGUT DE MIOCARDI	CORONARY	MIOCARDIAL INFARCTION
CIM10	I24	ALTRES MALALTIES ISQUÈMIQUES AGUDES DEL COR	CORONARY	ANGOR
CIM10	I24.0	TROMBOSI CORONÀRIA QUE NO PROVOCA UN INFART DE MIOCARDI	CORONARY	INDETERMINATE CORONARY ARTERY DISEASE

CIM10	I24.8	ALTRES FORMES DE MALALTIA ISQUÈMICA AGUDA DEL COR	CORONARY	INDETERMINATE CORONARY ARTERY DISEASE
CIM10	I24.9	MALALTIA ISQUÈMICA AGUDA DEL COR, NO ESPECIFICADA	CORONARY	ANGOR
CIM10	I25	MALALTIA ISQUÈMICA CRÒNICA DEL COR	CORONARY	INDETERMINATE CORONARY ARTERY DISEASE
CIM10	I25.0	MALALTIA CARDIOVASCULAR ATEROSCLERÒTICA, DESCRITA D'AQUESTA MANERA	CORONARY	INDETERMINATE CORONARY ARTERY DISEASE
CIM10	I25.1	MALALTIA ATEROSCLERÒTICA DEL COR	CORONARY	INDETERMINATE CORONARY ARTERY DISEASE
CIM10	I25.2	INFART ANTIC DE MIOCARDI	CORONARY	MIOCARDIAL INFARCTION
CIM10	I25.5	MIOCARDIOPATIA ISQUÈMICA	CORONARY	INDETERMINATE CORONARY ARTERY DISEASE
CIM10	I25.6	ISQUÈMIA SILENT DE MIOCARDI	CORONARY	INDETERMINATE CORONARY ARTERY DISEASE
CIM10	I25.8	ALTRES FORMES DE MALALTIA ISQUÈMICA CRÒNICA DEL COR	CORONARY	INDETERMINATE CORONARY ARTERY DISEASE
CIM10	I25.9	MALALTIA ISQUÈMICA CRÒNICA DEL COR, NO ESPECIFICADA	CORONARY	INDETERMINATE CORONARY ARTERY DISEASE
CIM10	I50.0	INSUFICIÈNCIA CARDÍACA CONGESTIVA	HEART FAILURE	HEART FAILURE_CONGESTIVE
CIM9	4280	Insuficiència cardíaca congestiva no especificada	HEART FAILURE	HEART FAILURE_CONGESTIVE
CIM9	42821	Insuficiència cardíaca sistòlica aguda	HEART FAILURE	HEART FAILURE_CONGESTIVE
CIM9	42831	Insuficiència cardíaca diastòlica aguda	HEART FAILURE	HEART FAILURE_CONGESTIVE
CIM9	42841	Insuficiència cardíaca combinada sistòlica/diastòlica aguda	HEART FAILURE	HEART FAILURE_CONGESTIVE
CIM10	I50	INSUFICIÈNCIA CARDÍACA	HEART FAILURE	HEART FAILURE_OTHERS
CIM10	I61	HEMORRÀGIA INTRAENCEFÀLICA	CEREBROVASCULAR	HEMORRHAGIC STROKE
CIM10	I61.0	HEMORRÀGIA INTRACEREBRAL DE L'HEMISFERI SUBCORTICAL	CEREBROVASCULAR	HEMORRHAGIC STROKE
CIM10	I61.1	HEMORRÀGIA INTRACEREBRAL DE L'HEMISFERI CORTICAL	CEREBROVASCULAR	HEMORRHAGIC STROKE
CIM10	I61.2	HEMORRÀGIA INTRACEREBRAL DE L'HEMISFERI, NO ESPECIFICADA	CEREBROVASCULAR	HEMORRHAGIC STROKE
CIM10	I61.3	HEMORRÀGIA INTRAENCEFÀLICA EN LA TIJA CEREBRAL	CEREBROVASCULAR	HEMORRHAGIC STROKE
CIM10	I61.4	HEMORRÀGIA INTRAENCEFÀLICA EN EL CEREBEL	CEREBROVASCULAR	HEMORRHAGIC STROKE
CIM10	I61.5	HEMORRÀGIA INTRAENCEFÀLICA, INTRAVENTRICULAR	CEREBROVASCULAR	HEMORRHAGIC STROKE
CIM10	I61.6	HEMORRÀGIA INTRAENCEFÀLICA DE LOCALITZACIONS MÚLTIPLES	CEREBROVASCULAR	HEMORRHAGIC STROKE
CIM10	I61.8	ALTRES HEMORRÀGIES INTRAENCEFÀLIQUES	CEREBROVASCULAR	HEMORRHAGIC STROKE
CIM10	I61.9	HEMORRÀGIA INTRAENCEFÀLICA, NO ESPECIFICADA	CEREBROVASCULAR	HEMORRHAGIC STROKE
CIM10	I63	INFART CEREBRAL	CEREBROVASCULAR	ISCHEMIC STROKE

CIM10	I63.0	INFART CEREBRAL SECUNDARI A TROMBOSI D’ARTÈRIES PRECEREBRALS	CEREBROVASCULAR	ISCHEMIC STROKE
CIM10	I63.1	INFART CEREBRAL SECUNDARI A EMBÒLIA D’ARTÈRIES PRECEREBRALS	CEREBROVASCULAR	ISCHEMIC STROKE
CIM10	I63.2	INFART CEREBRAL SECUNDARI A OCLUSIÓ O ESTENOSI NO ESPECIFICADA D’ARTÈRIES PRECEREBRALS	CEREBROVASCULAR	ISCHEMIC STROKE
CIM10	I63.3	INFART CEREBRAL SECUNDARI A TROMBOSI D’ARTÈRIES CEREBRALS	CEREBROVASCULAR	ISCHEMIC STROKE
CIM10	I63.4	INFART CEREBRAL SECUNDARI A EMBÒLIA D’ARTÈRIES CEREBRALS	CEREBROVASCULAR	ISCHEMIC STROKE
CIM10	I63.5	INFART CEREBRAL SECUNDARI A OCLUSIÓ O ESTENOSI NO ESPECIFICADA D’ARTÈRIES CEREBRALS	CEREBROVASCULAR	ISCHEMIC STROKE
CIM10	I63.6	INFART CEREBRAL SECUNDARI A TROMBOSI DE VENES CEREBRALS, NO PIOGEN	CEREBROVASCULAR	ISCHEMIC STROKE
CIM10	I63.8	ALTRES INFARTS CEREBRALS	CEREBROVASCULAR	ISCHEMIC STROKE
CIM10	I63.9	INFART CEREBRAL, NO ESPECIFICAT	CEREBROVASCULAR	ISCHEMIC STROKE
CIM10	I65	OCLUSIÓ I ESTENOSI D’ARTÈRIES PRECEREBRALS SENSE OCASIONAR INFART CEREBRAL	PERIPHERAL	CAROTID/CEREBRAL DISEASE
CIM10	I65.0	OCLUSIÓ I ESTENOSI L’ARTÈRIA VERTEBRAL	PERIPHERAL	CAROTID/CEREBRAL DISEASE
CIM10	I65.1	OCLUSIÓ I ESTENOSI DE L’ARTÈRIA BASILAR	PERIPHERAL	CAROTID/CEREBRAL DISEASE
CIM10	I65.2	OCLUSIÓ I ESTENOSI DE L’ARTÈRIA CARÒTIDA	PERIPHERAL	CAROTID/CEREBRAL DISEASE
CIM10	I65.3	OCLUSIÓ I ESTENOSI MÚLTIPLE BILATERAL D’ARTÈRIES PRECEREBRALS	PERIPHERAL	CAROTID/CEREBRAL DISEASE
CIM10	I65.8	OCLUSIÓ I ESTENOSI D’ALTRES ARTÈRIES PRECEREBRALS	PERIPHERAL	CAROTID/CEREBRAL DISEASE
CIM10	I65.9	OCLUSIÓ I ESTENOSI D’UNA ARTÈRIA PRECEREBRAL NO ESPECIFICADA	PERIPHERAL	CAROTID/CEREBRAL DISEASE
CIM10	I70.2	ATEROSCLEROSI DE LES ARTÈRIES DELS MEMBRES	PERIPHERAL	PERIPHERAL ARTERIAL DISEASE (PAD)
CIM10	I73.9	MALALTIA VASCULAR PERIFÈRICA, NO ESPECIFICADA	PERIPHERAL	PERIPHERAL ARTERIAL DISEASE (PAD)
CIM9	6	PROCEDIMENTS ENS VASOS SANGUINIS	PERIPHERAL	REVASCULARIZATION
CIM9	61	ANGIOPLÀSTIA PERCUTÀNIA DE VAS -OS EXTRACRANIAL -S	PERIPHERAL	REVASCULARIZATION
CIM9	62	ANGIPL PERCUTÀNIA O ATERECTO VAS INTRACEREBRAL	CEREBROVASCULAR	CEREBRAL REVASCULARIZATION
CIM9	360	ELIMINACIÓ OBSTRUCCIÓ ARTÈRIA CORONÀRIA I INSERCIÓ DE STENT	CORONARY	CORONARY REVASCULARIZATION
CIM9	361	ANASTOMOSI DE DESVIACIÓ PER A REVASCULARITZACIÓ CARDÍACA	CORONARY	CORONARY REVASCULARIZATION

CIM9	3610	DESVIACIÓ AORTOCORONÀRIA P/A REVASCULARITZACIÓ CARDÍACA,NSP	CORONARY	CORONARY REVASCULARIZATION
CIM9	362	REVASCULARITZACIÓ CARDÍACA P/IMPLANTACIÓ ARTERIAL;INDIRECTA	CORONARY	CORONARY REVASCULARIZATION
CIM9	363	ALTRES REVASCULARITZACIONS CARDÍAQUES	CORONARY	CORONARY REVASCULARIZATION
CIM9	3639	ALT.REVASCULARITZACIÓ CARDÍACA; ABRASIÓ EPICARDI	CORONARY	CORONARY REVASCULARIZATION
CIM9	3979	ALTRES TIPUS DE PROCEDIMENTS EN ALTRES VASOS	PERIPHERAL	REVASCULARIZATION
CIM9	3990	IMPL STENT NO ALLIBERADOR FÀRMACS VAS PERIFÈRIC NO CORONARI	PERIPHERAL	REVASCULARIZATION
CIM9	410	INFART DE MIOCARDI AGUT	CORONARY	MIOCARDIAL INFARCTION
CIM9	4100	INFART DE MIOCARDI AGUT DE PARET ANTEROLATERAL	CORONARY	MIOCARDIAL INFARCTION
CIM9	41000	INFART DE MIOCARDI AGUT DE PARET ANTEROLATERAL, EPISODI NO ESPECIFICAT	CORONARY	MIOCARDIAL INFARCTION
CIM9	41001	INFART DE MIOCARDI AGUT DE PARET ANTEROLATERAL, EPISODI INICIAL	CORONARY	MIOCARDIAL INFARCTION
CIM9	41002	INFART DE MIOCARDI AGUT DE PARET ANTEROLATERAL, EPISODI POSTERIOR	CORONARY	MIOCARDIAL INFARCTION
CIM9	4101	INFART AGUT MIOCARDI D’ALTRA PARET ANTERIOR	CORONARY	MIOCARDIAL INFARCTION
CIM9	41010	IAM D’ALTRA PARET ANTERIOR, ASSISTÈNCIA INESPECIFICADA	CORONARY	MIOCARDIAL INFARCTION
CIM9	41011	IAM D’ALTRA PARET ANTERIOR, ASSISTÈNCIA INICIAL	CORONARY	MIOCARDIAL INFARCTION
CIM9	41012	IAM D’ALTRA PARET ANTERIOR, ASSISTÈNCIA SUBSEGÜENT	CORONARY	MIOCARDIAL INFARCTION
CIM9	4102	INFART AGUT MIOCARDI, PARET INFEROLATERAL	CORONARY	MIOCARDIAL INFARCTION
CIM9	41020	IAM, PARET INFEROLATERAL, ASSISTÈNCIA INESPECIFICADA	CORONARY	MIOCARDIAL INFARCTION
CIM9	41021	IAM, PARET INFEROLATERAL, ASSISTÈNCIA INICIAL	CORONARY	MIOCARDIAL INFARCTION
CIM9	41022	INFART DE MIOCARDI AGUT DE LA PARET INFEROLATERAL, EPISODI POSTERIOR	CORONARY	MIOCARDIAL INFARCTION
CIM9	4103	INFART DE MIOCARDI AGUT DE LA PARET INFEROPOSTERIOR	CORONARY	MIOCARDIAL INFARCTION
CIM9	41030	INFART DE MIOCARDI AGUT DE LA PARET INFEROPOSTERIOR, EPISODI NO ESPECIFICAT	CORONARY	MIOCARDIAL INFARCTION
CIM9	41031	INFART DE MIOCARDI AGUT DE LA PARET INFEROPOSTERIOR, EPISODI INICIAL	CORONARY	MIOCARDIAL INFARCTION
CIM9	41032	INFART DE MIOCARDI AGUT DE LA PARET INFEROPOSTERIOR, EPISODI POSTERIOR	CORONARY	MIOCARDIAL INFARCTION
CIM9	4104	INFART DE MIOCARDI AGUT D’UNA ALTRA PART DE LA PARET INFERIOR	CORONARY	MIOCARDIAL INFARCTION

CIM9	41040	INFART DE MIOCARDI AGUT D’UNA ALTRA PART DE LA PARET INFERIOR, EPISODI NO ESPECIFICAT	CORONARY	MIOCARDIAL INFARCTION
CIM9	41041	INFART DE MIOCARDI AGUT D’UNA ALTRA PART DE LA PARET INFERIOR, EPISODI INICIAL	CORONARY	MIOCARDIAL INFARCTION
CIM9	41042	INFART DE MIOCARDI AGUT D’UNA ALTRA PART DE LA PARET INFERIOR, EPISODI POSTERIOR	CORONARY	MIOCARDIAL INFARCTION
CIM9	4105	INFART DE MIOCARDI AGUT D’UNA ALTRA PART DE LA PARET LATERAL	CORONARY	MIOCARDIAL INFARCTION
CIM9	41050	INFART DE MIOCARDI AGUT D’UNA ALTRA PART DE LA PARET LATERAL, EPISODI NO ESPECIFICAT	CORONARY	MIOCARDIAL INFARCTION
CIM9	41051	INFART DE MIOCARDI AGUT D’UNA ALTRA PART DE LA PARET LATERAL, EPISODI INICIAL	CORONARY	MIOCARDIAL INFARCTION
CIM9	41052	INFART DE MIOCARDI AGUT D’UNA ALTRA PART DE LA PARET LATERAL, EPISODI POSTERIOR	CORONARY	MIOCARDIAL INFARCTION
CIM9	4106	INFART DE MIOCARDI AGUT DE PARET POSTERIOR VERITABLE	CORONARY	MIOCARDIAL INFARCTION
CIM9	41060	INFART DE MIOCARDI AGUT DE PARET POSTERIOR VERITABLE, EPISODI NO ESPECIFICAT	CORONARY	MIOCARDIAL INFARCTION
CIM9	41061	INFART DE MIOCARDI AGUT DE PARET POSTERIOR VERITABLE, EPISODI INICIAL	CORONARY	MIOCARDIAL INFARCTION
CIM9	41062	INFART DE MIOCARDI AGUT DE PARET POSTERIOR VERITABLE, EPISODI POSTERIOR	CORONARY	MIOCARDIAL INFARCTION
CIM9	4107	INFART SUBENDOCARDÍAC AGUT	CORONARY	MIOCARDIAL INFARCTION
CIM9	41070	INFART SUBENDOCARDÍAC AGUT, EPISODI NO ESPECIFICAT	CORONARY	MIOCARDIAL INFARCTION
CIM9	41071	INFARTO SUBENDOCÁRDICO,EPISODIO DE ATENCIÓN INICIAL	CORONARY	MIOCARDIAL INFARCTION
CIM9	41072	INFART SUBENDOCARDÍAC AGUT, EPISODI POSTERIOR	CORONARY	MIOCARDIAL INFARCTION
CIM9	4108	INFART DE MIOCARDI AGUT D’ALTRES LOCALITZACIONS ESPECIFICADES	CORONARY	MIOCARDIAL INFARCTION
CIM9	41080	INFART DE MIOCARDI AGUT D’ALTRES LOCALITZACIONS ESPECIFICADES, EPISODI NO ESPECIFICAT	CORONARY	MIOCARDIAL INFARCTION
CIM9	41081	INFART DE MIOCARDI AGUT D’ALTRES LOCALITZACIONS ESPECIFICADES, EPISODI INICIAL	CORONARY	MIOCARDIAL INFARCTION
CIM9	41082	INFART DE MIOCARDI AGUT D’ALTRES LOCALITZACIONS ESPECIFICADES, EPISODI POSTERIOR	CORONARY	MIOCARDIAL INFARCTION
CIM9	4109	INFART DE MIOCARDI AGUT DE LOCALITZACIÓ NO ESPECIFICADA	CORONARY	MIOCARDIAL INFARCTION
CIM9	41090	INFART DE MIOCARDI AGUT DE LOCALITZACIÓ NO ESPECIFICADA, EPISODI NO ESPECIFICAT	CORONARY	MIOCARDIAL INFARCTION

CIM9	41091	INFART DE MIOCARDI AGUT DE LOCALITZACIÓ NO ESPECIFICADA, EPISODI INICIAL	CORONARY	MIOCARDIAL INFARCTION
CIM9	41092	INFART DE MIOCARDI AGUT DE LOCALITZACIÓ NO ESPECIFICADA, EPISODI POSTERIOR	CORONARY	MIOCARDIAL INFARCTION
CIM9	411	ALTRES FORMES AGUDES I SUBAGUDES DE CARDIOPATIA ISQUÈMICA	CORONARY	ANGOR
CIM9	4110	SÍNDROME POSTINFART DE MIOCARDI	CORONARY	MIOCARDIAL INFARCTION
CIM9	4111	SÍNDROME CORONÀRIA INTERMÈDIA	CORONARY	ANGOR
CIM9	4118	ALTRES FORMES AGUDES I SUBAGUDES DE CARDIOPATIA ISQUÈMICA	CORONARY	ANGOR
CIM9	41181	OCLUSIÓ CORONÀRIA AGUDA SENSE INFART DE MIOCARDI	CORONARY	ANGOR
CIM9	41189	ALTRES FORMES AGUDES I SUBAGUDES DE CARDIOPATIA ISQUÈMICA	CORONARY	ANGOR
CIM9	412	INFART ANTIC MIOCARDI; INFART MIOCARDI GUARIT DIAGNÒSTIC ECG	CORONARY	MIOCARDIAL INFARCTION
CIM9	413	ANGINA DE PIT	CORONARY	ANGOR
CIM9	4130	ANGINA DE DECÚBIT; ANGINA NOCTURNA	CORONARY	ANGOR
CIM9	4131	ANGINA DE PRINZMETAL; ANGINA DE PIT VARIANT	CORONARY	INDETERMINATE CORONARY ARTERY DISEASE
CIM9	4139	ALTRES TIPUS DÂ’ANGINA DE PIT I ANGINA DE PIT NO ESPECIFICADA	CORONARY	ANGOR
CIM9	414	ALTRES FORMES DE CARDIOPATIA ISQUÈMICA CRÒNICA	CORONARY	INDETERMINATE CORONARY ARTERY DISEASE
CIM9	41402	ATEROSCLEROSI CORONÀRIA D’EMPELT VENÓS AUTÒLEG	CORONARY	CORONARY REVASCULARIZATION
CIM9	41403	ATEROSCLEROSI CORONÀRIA D’EMPELT BIOLÒGIC NO AUTÒLEG	CORONARY	CORONARY REVASCULARIZATION
CIM9	41404	ATEROSCLEROSI CORONÀRIA D’EMPELT DE DERIVACIÓ ARTERIAL	CORONARY	CORONARY REVASCULARIZATION
CIM9	41405	ATEROSCLEROSI CORONÀRIA D’EMPELT DERIVACIÓ INESPECIFICAT;NOS	CORONARY	CORONARY REVASCULARIZATION
CIM9	41407	ATEROSCLEROSI EMPELT DERIVACIÓ (ARTERIAL,VENOSA) COR TRASPLA	CORONARY	CORONARY REVASCULARIZATION
CIM9	4148	ALTRES FORMES ESPECIFICADES DE CARDIOPATIA ISQUÈMICA CRÒNICA	CORONARY	INDETERMINATE CORONARY ARTERY DISEASE
CIM9	4149	CARDIOPATIA ISQUÈMICA CRÒNICA NO ESPECIFICADA	CORONARY	INDETERMINATE CORONARY ARTERY DISEASE
CIM10	I50.1	INSUFICIÈNCIA VENTRICULAR ESQUERRA	HEART FAILURE	HEART FAILURE_OTHERS

CIM10	I50.9	INSUFICIÈNCIA CARDÍACA, NO ESPECIFICADA	HEART FAILURE	HEART FAILURE_OTHERS
CIM10	I50.90	INSUFICIÈNCIA CARDÍACA, NO ESPECIFICADA	HEART FAILURE	HEART FAILURE_OTHERS
CIM10	I50.91	INSUFICIÈNCIA CARDÍACA, NO ESPECIFICADA	HEART FAILURE	HEART FAILURE_OTHERS
CIM9	428	INSUFICIÈNCIA CARDÍACA	HEART FAILURE	HEART FAILURE_OTHERS
CIM9	4281	INSUFICIÈNCIA CARDÍACA ESQUERRA; EDEMA AGUT DE PULMÓ	HEART FAILURE	HEART FAILURE_OTHERS
CIM9	4282	INSUFICIÈNCIA CARDÍACA SISTÒLICA	HEART FAILURE	HEART FAILURE_OTHERS
CIM9	42820	INSUFICIÈNCIA CARDÍACA SISTÒLICA INESPECIFICADA	HEART FAILURE	HEART FAILURE_OTHERS
CIM9	42822	INSUFICIÈNCIA CARDÍACA SISTÒLICA CRÒNICA	HEART FAILURE	HEART FAILURE_OTHERS
CIM9	42823	INSUFICIÈNCIA CARDÍACA SISTÒLICA CRÒNICA AGUDITZADA	HEART FAILURE	HEART FAILURE_OTHERS
CIM9	4283	INSUFICIÈNCIA CARDÍACA DIASTÒLICA	HEART FAILURE	HEART FAILURE_OTHERS
CIM9	42830	INSUFICIÈNCIA CARDÍACA DIASTÒLICA INESPECIFICADA	HEART FAILURE	HEART FAILURE_OTHERS
CIM9	42832	INSUFICIÈNCIA CARDÍACA DIASTÒLICA CRÒNICA	HEART FAILURE	HEART FAILURE_OTHERS
CIM9	42833	INSUFICIÈNCIA CARDÍACA DIASTÒLICA CRÒNICA AGUDITZADA	HEART FAILURE	HEART FAILURE_OTHERS
CIM9	4284	INSUFICIÈNCIA CARDÍACA COMBINADA, SISTÒLICA I DIASTÒLICA	HEART FAILURE	HEART FAILURE_OTHERS
CIM9	42840	INSUFICIÈNCIA CARDÍACA COMBINADA SISTÒLICA/DIASTÒLICA INESP.	HEART FAILURE	HEART FAILURE_OTHERS
CIM9	42842	INSUFICIÈNCIA CARDÍACA COMBINADA SISTÒLICA/DIASTÒLICA CRÒNIC	HEART FAILURE	HEART FAILURE_OTHERS
CIM9	42843	INSUFICIÈNCIA CARDÍACA COMBINADA SISTÒ./DIASTÒ.CRÒN.AGUDITZ.	HEART FAILURE	HEART FAILURE_OTHERS
CIM9	4289	INSUFICIÈNCIA CARDÍACA INESP.; NOS: CARDÍACA, MIOCARDÍACA	HEART FAILURE	HEART FAILURE_OTHERS
CIM9	4297	DETERMINADES SEQÜELES D'INFART DE MIOCARDI NO CLASSIFICADES A CAP ALTRE LLOC	CORONARY	MIOCARDIAL INFARCTION
CIM9	42971	DEFECTE SEPTAL CARDÍAC ADQUIRIT POSTERIOR A INFART DE MIOCARDI	CORONARY	MIOCARDIAL INFARCTION
CIM9	42979	ALTRES SEQÜELES D'INFART DE MIOCARDI NO CLASSIFICADES A CAP ALTRE LLOC	CORONARY	MIOCARDIAL INFARCTION
CIM9	431	HEMORRÀGIA INTRACEREBRAL	CEREBROVASCULAR	HEMORRHAGIC STROKE
CIM9	432	ALTRES HEMORRÀGIES INTRACRANIALS I HEMORRÀGIES INTRACRANIALS NO ESPECIFICADES	CEREBROVASCULAR	HEMORRHAGIC STROKE
CIM9	433	OCLUSIÓ I ESTENOSI D'ARTÈRIES PRECEREBRALS	PERIPHERAL	CAROTID/CEREBRAL DISEASE
CIM9	4330	OCLUSIÓ I ESTENOSI ARTÈRIA BASILAR	PERIPHERAL	CAROTID/CEREBRAL DISEASE
CIM9	43300	OCLUSIÓ I ESTENOSI ARTÈRIA BASILAR, SENSE INFART CEREBRAL	PERIPHERAL	CAROTID/CEREBRAL DISEASE
CIM9	43301	OCLUSIÓ I ESTENOSI ARTÈRIA BASILAR, AMB INFART CEREBRAL	CEREBROVASCULAR	ISCHEMIC STROKE
CIM9	4331	OCLUSIÓ I ESTENOSI ARTÈRIA CARÒTIDE	PERIPHERAL	CAROTID/CEREBRAL DISEASE

CIM9	43310	OCLUSIÓ I ESTENOSI ARTÈRIA CARÒTIDE, SENSE INFART CEREBRAL	PERIPHERAL	CAROTID/CEREBRAL DISEASE
CIM9	43311	OCLUSIÓ I ESTENOSI ARTÈRIA CARÒTIDE, AMB INFART CEREBRAL	CEREBROVASCULAR	ISCHEMIC STROKE
CIM9	4332	OCLUSIÓ I ESTENOSI ARTÈRIA VERTEBRAL	PERIPHERAL	CAROTID/CEREBRAL DISEASE
CIM9	43320	OCLUSIÓ I ESTENOSI ARTÈRIA VERTEBRAL, SENSE INFART CEREBRAL	PERIPHERAL	CAROTID/CEREBRAL DISEASE
CIM9	43321	OCLUSIÓ I ESTENOSI ARTÈRIA VERTEBRAL, AMB INFART CEREBRAL	CEREBROVASCULAR	ISCHEMIC STROKE
CIM9	4333	OCLUSIÓ I ESTENOSI MÚLTIPLE/BILATERAL ARTÈRIES PRECEREBRALS	PERIPHERAL	CAROTID/CEREBRAL DISEASE
CIM9	43330	OCLUSIÓ/ESTENOSI MÚLT./BILAT.ART.PRECEREBRALS,S/INFART CERV.	PERIPHERAL	CAROTID/CEREBRAL DISEASE
CIM9	43331	OCLUSIÓ/ESTENOSI MÚLT./BILAT.ART.PRECEREBRALS,A/INFART CERV.	CEREBROVASCULAR	ISCHEMIC STROKE
CIM9	4338	OCLUSIÓ I ESTENOSI ALTR.ARTÈRIES PRECEREBRALS ESPECIFICADES	PERIPHERAL	CAROTID/CEREBRAL DISEASE
CIM9	43380	OCLUSIÓ/ESTENOSI ALT.ART.PRECEREBRALS ESP.,S/INFART CERVELL	PERIPHERAL	CAROTID/CEREBRAL DISEASE
CIM9	43381	OCLUSIÓ/ESTENOSI ALT.ART.PRECEREBRALS ESP.,A/INFART CERVELL	CEREBROVASCULAR	ISCHEMIC STROKE
CIM9	4339	OCLUSIÓ I ESTENOSI ARTÈRIA PRECEREBRAL INESP.; NOS	PERIPHERAL	CAROTID/CEREBRAL DISEASE
CIM9	43390	OCLUSIÓ/ESTENOSI ARTÈRIA PRECEREBRAL INESP.,S/INFART CERVELL	PERIPHERAL	CAROTID/CEREBRAL DISEASE
CIM9	43391	OCLUSIÓ/ESTENOSI ARTÈRIA PRECEREBRAL INESP.,A/INFART CERVELL	CEREBROVASCULAR	ISCHEMIC STROKE
CIM9	434	OCLUSIÓ D’ARTÈRIES CEREBRALS	PERIPHERAL	CAROTID/CEREBRAL DISEASE
CIM9	4340	TROMBOSI CEREBRAL; TROMBOSI D’ARTÈRIES CEREBRALS	CEREBROVASCULAR	ISCHEMIC STROKE
CIM9	43400	TROMBOSI CEREBRAL SENSE MENCIÓ D’INFART CEREBRAL	CEREBROVASCULAR	ISCHEMIC STROKE
CIM9	43401	TROMBOSI CEREBRAL AMB INFART CEREBRAL	CEREBROVASCULAR	ISCHEMIC STROKE
CIM9	4341	EMBOLISME CEREBRAL	CEREBROVASCULAR	ISCHEMIC STROKE
CIM9	43410	EMBOLISME CEREBRAL, SENSE INFART CEREBRAL	CEREBROVASCULAR	ISCHEMIC STROKE
CIM9	43411	EMBOLISME CEREBRAL, AMB INFART CEREBRAL	CEREBROVASCULAR	ISCHEMIC STROKE
CIM9	4349	OCLUSIÓ ARTÈRIA CEREBRAL INESPECIFICADA	CEREBROVASCULAR	ISCHEMIC STROKE
CIM9	43490	OCLUSIÓ D’ARTÈRIA CEREBRAL NO ESPECIFICADA SENSE MENCIÓ D’INFART CEREBRAL	CEREBROVASCULAR	ISCHEMIC STROKE
CIM9	43491	OCLUSIÓ D’ARTÈRIA CEREBRAL NO ESPECIFICADA AMB INFART CEREBRAL	CEREBROVASCULAR	ISCHEMIC STROKE

CIM9	435	ISQUÈMIA CEREBRAL TRANSITÒRIA	CEREBROVASCULAR	TRANSIENT ISCHEMIC ATTACK (TIA)
CIM9	4350	SÍNDROME DE L’ARTÈRIA BASILAR	CEREBROVASCULAR	TRANSIENT ISCHEMIC ATTACK (TIA)
CIM9	4351	SÍNDROME DE L’ARTÈRIA VERTEBRAL	CEREBROVASCULAR	TRANSIENT ISCHEMIC ATTACK (TIA)
CIM9	4352	SÍNDROME DEL FURT DE LA SUBCLÀVIA	CEREBROVASCULAR	TRANSIENT ISCHEMIC ATTACK (TIA)
CIM9	4353	SÍNDROME DE L’ARTÈRIA VERTEBROBASILAR	CEREBROVASCULAR	TRANSIENT ISCHEMIC ATTACK (TIA)
CIM9	4358	ALTRES ISQUÈMIES CEREBRALS TRANSITÒRIES ESPECIFICADES	CEREBROVASCULAR	TRANSIENT ISCHEMIC ATTACK (TIA)
CIM9	4359	ISQUÈMIA CEREBRAL TRANSITÒRIA NO ESPECIFICADA	CEREBROVASCULAR	TRANSIENT ISCHEMIC ATTACK (TIA)
CIM9	436	MALALTIA CEREBROVASCULAR AGUDA MAL DEFINIDA; APOPLEXIA	CEREBROVASCULAR	ISCHEMIC STROKE
CIM9	4377	AMNÈSIA GLOBAL TRANSITÒRIA	CEREBROVASCULAR	TRANSIENT ISCHEMIC ATTACK (TIA)
CIM9	44021	ATEROSCLEROSI D’EXTREMITATS AMB CLAUDICACIÓ INTERMITENT	PERIPHERAL	PERIPHERAL ARTERIAL DISEASE (PAD)
CIM9	44022	ATEROSCLEROSI D’EXTREMITATS AMB DOLOR DE REPÒS	PERIPHERAL	PERIPHERAL ARTERIAL DISEASE (PAD)
CIM9	44023	ATEROSCLEROSI D’EXTREMITATS AMB ULCERACIÓ	PERIPHERAL	PERIPHERAL ARTERIAL DISEASE (PAD)
CIM9	44024	ATEROSCLEROSI D’EXTREMITATS AMB GANGRENA	PERIPHERAL	PERIPHERAL ARTERIAL DISEASE (PAD)
CIM9	4403	ATEROSCLEROSI EN EMPELT DE DERIVACIÓ DE LES EXTREMITATS	PERIPHERAL	REVASCULARIZATION
CIM9	44030	ATEROSCLEROSI D’EMPELT DE DERIVACIÓ DE LES EXTREMITATS NO ESPECIFICAT	PERIPHERAL	REVASCULARIZATION
CIM9	44031	ATEROSCLEROSI D’EMPELT DE DERIVACIÓ DE LES EXTREMITATS VENÓS AUTÒLEG	PERIPHERAL	REVASCULARIZATION
CIM9	44032	ATEROSCLEROSI D’EMPELT DE DERIVACIÓ DE LES EXTREMITATS BIOLÒGIC NO AUTÒLEG	PERIPHERAL	REVASCULARIZATION
CIM9	4404	OCLUSIÓ TOTAL CRÒNICA D’ARTÈRIA DE LES EXTREMITATS	PERIPHERAL	PERIPHERAL ARTERIAL DISEASE (PAD)
CIM9	4439	MAL.VASCULAR PERIFÈRICA INESP.; CLAUDICACIÓ INTERMITENT	PERIPHERAL	PERIPHERAL ARTERIAL DISEASE (PAD)
CIM9	V4581	ESTAT DE DERIVACIÓ AORTOCORONÀRIA	CORONARY	CORONARY REVASCULARIZATION
CIM9	V4582	ESTAT D’ANGIOPLÀSTIA CORONÀRIA TRANSLUMINAL PERCUTÀNIA	CORONARY	CORONARY REVASCULARIZATION

Table S3. Population characteristics at baseline by age group.

	Overall	Y (< 35 y)	EA (35 - 55/60 y)	MA (55/60 – 65 y)	YO (65 – 75 y)	MVO (> 75 y)
	**(N=3769563)**	**(N=561847, 14.9%)**	**(N=1986287, 52.7%)**	**(N=439931, 11.7%)**	**(N=418105, 11.1%)**	**(N=363393, 9.6%)**
**Sex**						
Women	1949847 (51.7%)	271639 (48.3%)	1053464 (53.0%)	157601 (35.8%)	234879 (56.2%)	232264 (63.9%)
Men	1819716 (48.3%)	290208 (51.7%)	932823 (47.0%)	282330 (64.2%)	183226 (43.8%)	131129 (36.1%)
**Age (Years)**						
Mean (SD)	51.2 (15.2)	32.5 (1.43)	45.1 (6.61)	60.7 (2.77)	69.7 (2.98)	80.9 (4.02)
Median [Min, Max]	48.3 [30.0, 89.9]	32.6 [30.0, 34.9]	44.5 [35.0, 59.9]	61.2 [55.0, 64.9]	69.5 [65.0, 74.9]	80.3 [75.0, 89.9]
[Q1-Q3]	[38.3-62.1]	[31.3-33.8]	[39.4-50.3]	[58.6-62.9]	[67.0-72.4]	[77.4-83.9]
**Body mass index**	28.5 (5.07)	26.3 (5.23)	28.2 (5.49)	29.3 (4.72)	29.4 (4.64)	28.6 (4.49)
Missing	3079323 (81.7%)	503244 (89.6%)	1734161 (87.3%)	331810 (75.4%)	270568 (64.7%)	239540 (65.9%)
**Obesity (BMI ≥ 30kg/m^2)**	42098 (34.2%)	235815 (20.7%)	12107 (31.8%)	80176 (39.2%)	42366 (40%)	59068 (34%)
Missing	239540 (65.9%)	3079323 (81.7%)	503244 (89.6%)	1734161 (87.3%)	331810 (75.4%)	270568 (64.7%)
**Smoking**						
No smoker	1213799 (32.2%)	112429 (20.0%)	497626 (25.1%)	166053 (37.7%)	225346 (53.9%)	212345 (58.4%)
Smoker	738122 (19.6%)	133888 (23.8%)	464082 (23.4%)	81524 (18.5%)	41656 (10.0%)	16972 (4.7%)
Former smoker	280079 (7.4%)	24727 (4.4%)	127730 (6.4%)	50467 (11.5%)	45821 (11.0%)	31334 (8.6%)
Missing	1537563 (40.8%)	290803 (51.8%)	896849 (45.2%)	141887 (32.3%)	105282 (25.2%)	102742 (28.3%)
**Type 2 diabetes**	247751 (6.6%)	1204 (0.2%)	51295 (2.6%)	52636 (12.0%)	72655 (17.4%)	69961 (19.3%)
**Type 1 diabetes**	9372 (0.2%)	1905 (0.3%)	5731 (0.3%)	822 (0.2%)	553 (0.1%)	361 (0.1%)
**Hypertension**	735031 (19.5%)	5947 (1.1%)	173255 (8.7%)	141155 (32.1%)	202264 (48.4%)	212410 (58.5%)
**Systolic Blood Pressure**	130 (15.8)	120 (13.5)	126 (14.9)	133 (14.7)	135 (14.7)	136 (15.6)
Missing	2585627 (68.6%)	470906 (83.8%)	1536994 (77.4%)	252823 (57.5%)	177060 (42.3%)	147844 (40.7%)

**Diastolic Blood Pressure**	76.6 (9.63)	72.7 (9.73)	77.6 (10.0)	79.0 (9.02)	76.7 (8.73)	74.0 (9.07)
Missing	2585627 (68.6%)	470906 (83.8%)	1536994 (77.4%)	252823 (57.5%)	177060 (42.3%)	147844 (40.7%)
**Atherogenic dyslipidemia**	96518 (2.6%)	3606 (0.6%)	39720 (2.0%)	17679 (4.0%)	19964 (4.8%)	15549 (4.3%)
No	739048 (19.6%)	34899 (6.2%)	278380 (14.0%)	132531 (30.1%)	162618 (38.9%)	130620 (35.9%)
Missing	2933997 (77.8%)	523342 (93.1%)	1668187 (84.0%)	289721 (65.9%)	235523 (56.3%)	217224 (59.8%)
**Total cholesterol**	209 (38.5)	189 (36.5)	210 (39.2)	215 (37.8)	211 (36.7)	205 (37.5)
Missing	2600417 (69.0%)	477539 (85.0%)	1498285 (75.4%)	252488 (57.4%)	194212 (46.5%)	177893 (49.0%)
**HDLc**	55.4 (14.8)	54.5 (15.3)	55.3 (15.2)	54.3 (14.4)	56.0 (14.3)	56.3 (14.6)
Missing	2901888 (77.0%)	518620 (92.3%)	1649482 (83.0%)	286807 (65.2%)	232347 (55.6%)	214632 (59.1%)
**Triglycerides**	132 (88.4)	119 (95.0)	136 (105)	140 (90.8)	129 (67.1)	123 (60.2)
Missing	2843538 (75.4%)	514363 (91.5%)	1627011 (81.9%)	276759 (62.9%)	221855 (53.1%)	203550 (56.0%)
**Statins**	457930 (12.1%)	3652 (0.7%)	114988 (5.8%)	98136 (22.3%)	134282 (32.1%)	106872 (29.4%)
**BP lowering treatment**	781084 (20.7%)	8288 (1.5%)	187773 (9.5%)	148401 (33.7%)	214106 (51.2%)	222516 (61.2%)
**Family history of CVD**	11397 (0.3%)	1268 (0.2%)	7129 (0.4%)	1566 (0.4%)	1024 (0.2%)	410 (0.1%)
**Chronic kidney disease**	48500 (1.3%)	685 (0.1%)	7300 (0.4%)	6053 (1.4%)	11603 (2.8%)	22859 (6.3%)
**UACR**	22.3 (101)	14.8 (82.4)	18.7 (100)	19.9 (91.9)	20.4 (91.4)	31.1 (120)
Missing	3571997 (94.8%)	558340 (99.4%)	1933972 (97.4%)	399808 (90.9%)	362682 (86.7%)	317195 (87.3%)
**Glomerular Filtration (CKD-EPI)**	79.9 (13.5)	88.3 (4.91)	85.6 (8.00)	81.0 (11.1)	75.7 (13.6)	65.5 (16.2)
Missing	2617524 (69.4%)	479008 (85.3%)	1508965 (76.0%)	256020 (58.2%)	196541 (47.0%)	176990 (48.7%)
**Alcohol consumption (Level of risk)**						
No risk	473347 (12.6%)	36014 (6.4%)	182641 (9.2%)	64249 (14.6%)	97577 (23.3%)	92866 (25.6%)
Low risk	206190 (5.5%)	14040 (2.5%)	80448 (4.1%)	40951 (9.3%)	43825 (10.5%)	26926 (7.4%)
High risk	20317 (0.5%)	1327 (0.2%)	9389 (0.5%)	4858 (1.1%)	3370 (0.8%)	1373 4%)
Missing	3069709 (81.4%)	510466 (90.9%)	1713809 (86.3%)	329873 (75.0%)	273333 (65.4%)	242228 (66.7%)

Data are mean ± standard deviation, n (%) or median (Q1-Q3) as appropriate. p<0.001 for all characteristics through age groups.

Y: young (< 35 y); EA: early adulthood (35-55/60 (men/women)y); MA: middle adulthood (55/60-65 (men/women)y); YO: young old (65-75 y); and MVO: middle-to-very old (> 75 y). HDLc: high-density lipoprotein cholesterol; BP: blood pressure; CVD: cardiovascular disease; UACR: urine albumin-to-creatinine ratio

Table S4. First manifestation of cardiovascular disease within each territory (coronary, heart failure, cerebrovascular, and peripheral territories) in the entire population, and in women and men separately

Manifestation	**Sex**	**[ALL]**	**Y (<35 y)**	**EA (35 – 55/60 y)**	**MA (55/60 – 65 y)**	**YO (65 – 75 y)**	**MVO (> 75 y)**
	**Entire population**	N=251111	N=2361	N=48722	N=41974	N=63666	N=94388
	Women	N=115055	N=968	N=19198	N=9853	N=28565	N=56471
	Men	N=136056	N=1393	N=29524	N=32121	N=35101	N=37917
**Heart Failure**							
**Congestive/acute heart failure**	**Entire population**	**31788 (12.7%)**	**133 (5.63%)**	**2812 (5.77%)**	**2847 (6.78%)**	**6928 (10.9%)**	**19068 (20.2%)**
	Women	18363 (16.0%)	51 (5.27%)	1345 (7.01%)	839 (8.52%)	3707 (13.0%)	12421 (22.0%)
	Men	13425 (9.87%)	82 (5.89%)	1467 (4.97%)	2008 (6.25%)	3221 (9.18%)	6647 (17.5%)
**Heart failure others**	**Entire population**	**41068 (16.4%)**	**174 (7.37%)**	**3653 (7.50%)**	**4141 (9.87%)**	**10274 (16.1%)**	**22826 (24.2%)**
	Women	24716 (21.5%)	66 (6.82%)	1770 (9.22%)	1467 (14.9%)	5978 (20.9%)	15435 (27.3%)
	Men	16352 (12.0%)	108 (7.75%)	1883 (6.38%)	2674 (8.32%)	4296 (12.2%)	7391 (19.5%)
**Peripheral territories**							
**Peripheral Arterial disease (PAD)**	**Entire population**	**33847 (13.5%)**	**194 (8.22%)**	**7307 (15.0%)**	**7661 (18.3%)**	**9303 (14.6%)**	**9382 (9.94%)**
	Women	9821 (8.54%)	71 (7.33%)	2219 (11.6%)	927 (9.41%)	2500 (8.75%)	4104 (7.27%)
	Men	24026 (17.7%)	123 (8.83%)	5088 (17.2%)	6734 (21.0%)	6803 (19.4%)	5278 (13.9%)
**Peripheral carotid/cerebral disease**	**Entire population**	**6663 (2.65%)**	**22 (0.93%)**	**1081 (2.22%)**	**1373 (3.27%)**	**2246 (3.53%)**	**1941 (2.06%)**
	Women	2847 (2.47%)	11 (1.14%)	591 (3.08%)	385 (3.91%)	931 (3.26%)	929 (1.65%)
	Men	3816 (2.80%)	11 (0.79%)	490 (1.66%)	988 (3.08%)	1315 (3.75%)	1012 (2.67%)

**Peripheral revascularization**	**Entire population**	**3054 (1.22%)**	**159 (6.73%)**	**1171 (2.40%)**	**618 (1.47%)**	**678 (1.06%)**	**428 (0.45%)**
	Women	1246 (1.08%)	89 (9.19%)	618 (3.22%)	137 (1.39%)	227 (0.79%)	175 (0.31%)
	Men	1808 (1.33%)	70 (5.03%)	553 (1.87%)	481 (1.50%)	451 (1.28%)	253 (0.67%)
**Cerebrovascular disease**							
**Transient Ischemic Attack**	**Entire population**	**24063 (9.58%)**	**286 (12.1%)**	**4931 (10.1%)**	**4120 (9.82%)**	**6275 (9.86%)**	**8451 (8.95%)**
	Women	13368 (11.6%)	151 (15.6%)	2865 (14.9%)	1573 (16.0%)	3406 (11.9%)	5373 (9.51%)
	Men	10695 (7.86%)	135 (9.69%)	2066 (7.00%)	2547 (7.93%)	2869 (8.17%)	3078 (8.12%)
**Ischemic stroke**	**Entire population**	**31818 (12.7%)**	**310 (13.1%)**	**5710 (11.7%)**	**4834 (11.5%)**	**8485 (13.3%)**	**12479 (13.2%)**
	Women	15158 (13.2%)	148 (15.3%)	2403 (12.5%)	1165 (11.8%)	3941 (13.8%)	7501 (13.3%)
	Men	16660 (12.2%)	162 (11.6%)	3307 (11.2%)	3669 (11.4%)	4544 (12.9%)	4978 (13.1%)
**Hemorrhagic stroke**	**Entire population**	**6481 (2.58%)**	**146 (6.18%)**	**1745 (3.58%)**	**937 (2.23%)**	**1520 (2.39%)**	**2133 (2.26%)**
	Women	2975 (2.59%)	64 (6.61%)	848 (4.42%)	212 (2.15%)	652 (2.28%)	1199 (2.12%)
	Men	3506 (2.58%)	82 (5.89%)	897 (3.04%)	725 (2.26%)	868 (2.47%)	934 (2.46%)
**Cerebral revascularization**	**Entire population**	**127 (0.05%)**	**5 (0.21%)**	**72 (0.15%)**	**16 (0.04%)**	**28 (0.04%)**	**6 (0.01%)**
	Women	76 (0.07%)	2 (0.21%)	47 (0.24%)	8 (0.08%)	15 (0.05%)	4 (0.01%)
	Men	51 (0.04%)	3 (0.22%)	25 (0.08%)	8 (0.02%)	13 (0.04%)	2 (0.01%)
**Coronary disease**							
**Coronary indeterminate heart disease**	**Entire population**	**24576 (9.79%)**	**337 (14.3%)**	**5548 (11.4%)**	**5237 (12.5%)**	**6681 (10.5%)**	**6773 (7.18%)**
	Women	9777 (8.50%)	158 (16.3%)	2184 (11.4%)	1184 (12.0%)	2719 (9.52%)	3532 (6.25%)
	Men	14799 (10.9%)	179 (12.8%)	3364 (11.4%)	4053 (12.6%)	3962 (11.3%)	3241 (8.55%)
**Angor**	**Entire population**	**20218 (8.05%)**	**167 (7.07%)**	**5045 (10.4%)**	**4425 (10.5%)**	**5543 (8.71%)**	**5038 (5.34%)**
	Women	8668 (7.53%)	59 (6.10%)	2049 (10.7%)	1174 (11.9%)	2544 (8.91%)	2842 (5.03%)
	Men	11550 (8.49%)	108 (7.75%)	2996 (10.1%)	3251 (10.1%)	2999 (8.54%)	2196 (5.79%)

**Myocardial infarction**	**Entire population**	**26871 (10.7%)**	**419 (17.7%)**	**9559 (19.6%)**	**5688 (13.6%)**	**5528 (8.68%)**	**5677 (6.01%)**
	Women	7871 (6.84%)	91 (9.40%)	2217 (11.5%)	769 (7.80%)	1907 (6.68%)	2887 (5.11%)
	Men	19000 (14.0%)	328 (23.5%)	7342 (24.9%)	4919 (15.3%)	3621 (10.3%)	2790 (7.36%)
**Coronary revascularization**	**Entire population**	**537 (0.21%)**	**9 (0.38%)**	**88 (0.18%)**	**77 (0.18%)**	**177 (0.28%)**	**186 (0.20%)**
	Women	169 (0.15%)	7 (0.72%)	42 (0.22%)	13 (0.13%)	38 (0.13%)	69 (0.12%)
	Men	368 (0.27%)	2 (0.14%)	46 (0.16%)	64 (0.20%)	139 (0.40%)	117 (0.31%)

Data indicate number and percentage. p<0.001through age groups for the entire population, men and women.

EA, early adulthood, 35-55/60 y (men/women); **MA, middle adulthood**, 55/60-65 y (men/women); **MVO, middle-to-very old**, > 75 years; **Y, young**, < 35 y and **YO, young old**, 65-75 y.

Figure S2. First subtype of manifestations registered within each type of cardiovascular disease (coronary, heart failure, cerebrovascular, and peripheral territories) in the entire population and in women and men separately.

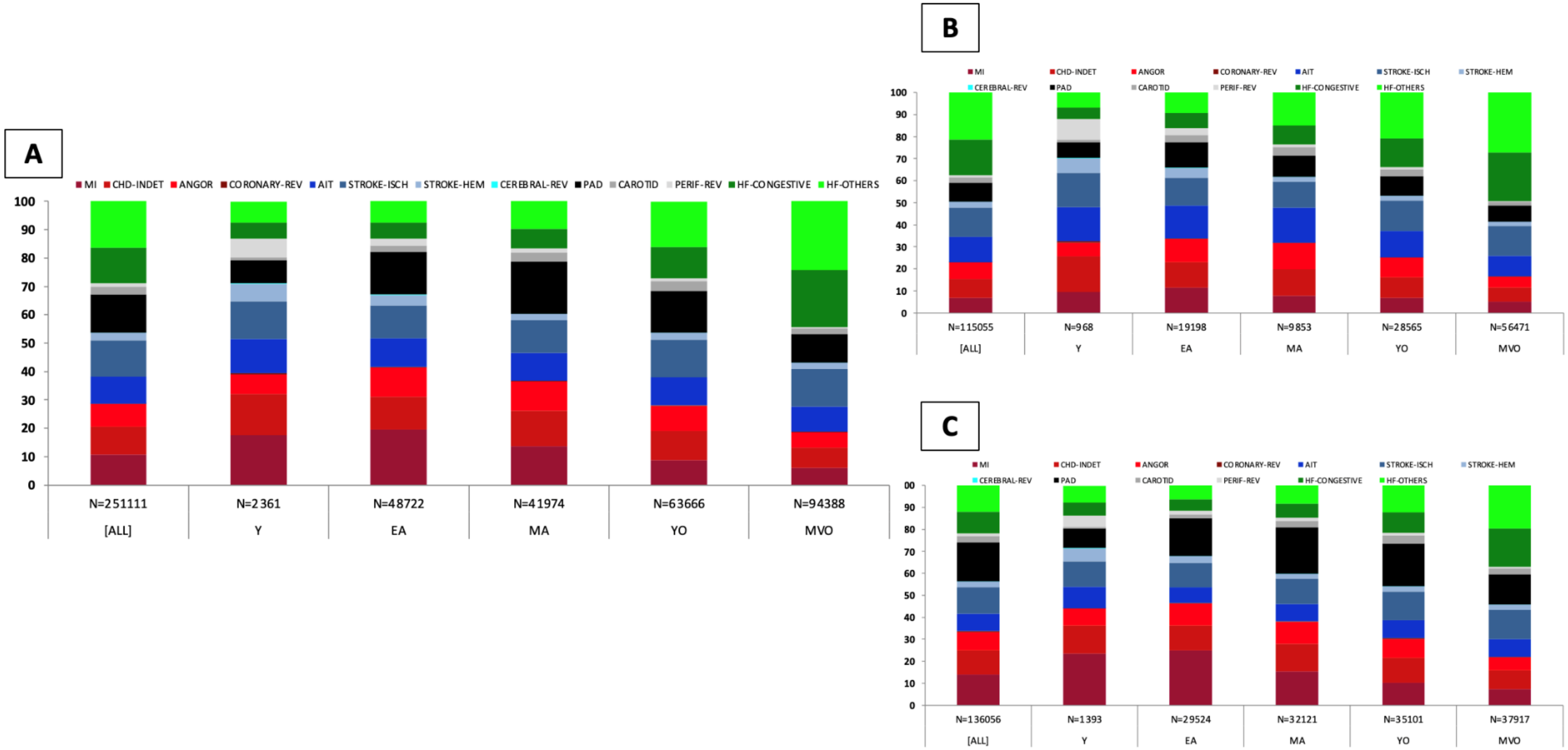

Figure A, entire population; Figure B, women; Figure C, men.

Y, young, < 35 y; **EA, early adulthood**, 35-55/60 y (men/women); **MA, middle adulthood**, 55/60-65 (men/women); **YO, young old**,65-75 y; and **MVO, middle-to-very old**, > 75 years.

MI: myocardial infarction; CHD-INDET: indetermined coronary heart disease; CORONARY-REVAS: coronary revascularization (including coronary-bypass or stenting); AIT: transient ischemic attack; STROKE-ISCH: ischemic stroke; STROKE-HEM: hemorrhagic stroke; CEREBRAL-REVAS: cerebral revascularization; PAD: peripheral artery disease; CAROTID: peripheral carotid disease; PERIPHERAL-REVAS: peripheral revascularization; HF-CONGESTIVE: congestive/acute heart failure; HF-OTHERS: others diagnostic of heart failure.

Table S5. Baseline population characteristics (F, female and M, men) and hazard ratio (95% confidence interval) according to the occurrence of a first cardiovascular event during follow-up.

	**Y** (<35 y)			**EA** (35 – 55/60 y)			**MA** (55/60 – 65 y)			**YO** (65 – 75 y)			**MVO** (> 75 y)		
	No event	Event	HR	No event	Event	HR	No event	Event	HR	No event	Event	HR	No event	Event	HR
**Number** F	N=270671	N=968		N=1034266	N=19198		N=147748	N=9853		N=206314	N=28565		N=175793	N=56471	
M	N=288815	N=1393		N=903299	N=29524		N=250209	N=32121		N=148125	N=35101		N=93212	N=37917	
**Age (years)** F	32.5 (1.43)	32.7 (1.44)	**1.07 [1.02; 1.12]**	46.2 (7.14)	51.0 (6.50)	**1.10 [1.10; 1.10]**	62.4 (1.43)	62.6 (1.44)	**1.09 [1.07; 1.10]**	69.6 (2.98)	70.5 (2.94)	**1.10 [1.09; 1.10]**	81.0 (4.06)	81.6 (4.03)	**1.05 [1.05; 1.06]**
M	32.5 (1.43)	32.8 (1.41)	**1.17 [1.12; 1.21]**	43.8 (5.65)	47.8 (5.11)	**1.14 [1.13; 1.14]**	59.7 (2.88)	60.2 (2.86)	**1.07 [1.06; 1.07]**	69.5 (2.95)	70.1 (2.97)	**1.06 [1.06; 1.07]**	80.4 (3.91)	80.7 (3.88)	**1.04 [1.04; 1.04]**
**Smoker** F	59265 (42.9%)	286 (45.3%)	**1.10 [0.94; 1.30]**	213072 (36.0%)	5811 (41.7%)	**1.29 [1.25; 1.34]**	11359 (11.0%)	1178 (15.1%)	**1.47 [1.38; 1.56]**	7514 (4.91%)	1374 (5.90%)	**1.21 [1.15; 1.28]**	2471 (2.01%)	848 (1.95%)	**1.00 [0.93; 1.07]**
M	73808 (56.1%)	529 (64.9%)	**1.49 [1.27; 1.74]**	232040 (50.1%)	13159 (65.5%)	**2.34 [2.26; 2.43]**	57796 (35.6%)	11191 (46.3%)	**1.79 [1.74; 1.84]**	24740 (22.9%)	8028 (28.5%)	**1.51 [1.47; 1.56]**	9347 (14.4%)	4306 (14.7%)	**1.12 [1.08; 1.16]**
**T2DM** F	532 (0.20%)	8 (0.83%)	**4.21 [2.10; 8.44]**	23136 (2.24%)	2263 (11.8%)	**5.69 [5.44; 5.94]**	13783 (9.33%)	2124 (21.6%)	**2.59 [2.46; 2.71]**	28519 (13.8%)	7594 (26.6%)	**2.16 [2.10; 2.22]**	28850 (16.4%)	13596 (24.1%)	**1.58 [1.55; 1.61]**
M	641 (0.22%)	23 (1.65%)	**7.48 [4.96; 11.3]**	22387 (2.48%)	3509 (11.9%)	**5.08 [4.90; 5.26]**	29126 (11.6%)	7603 (23.7%)	**2.24 [2.18; 2.29]**	26218 (17.7%)	10324 (29.4%)	**1.83 [1.79; 1.87]**	17407 (18.7%)	10108 (26.7%)	**1.53 [1.50; 1.57]**
**T1DM** F	722 (0.27%)	15 (1.55%)	**5.85 [3.51; 9.75]**	2107 (0.20%)	176 (0.92%)	**4.45 [3.84; 5.16]**	136 (0.09%)	25 (0.25%)	**2.63 [1.78; 3.89]**	158 (0.08%)	53 (0.19%)	**2.37 [1.81; 3.10]**	109 (0.06%)	58 (0.10%)	**1.82 [1.41; 2.36**
M	1052 (0.36%)	21 (1.51%)	**4.18 [2.71; 6.43]**	2645 (0.29%)	303 (1.03%)	**3.44 [3.07; 3.85**	412 (0.16%)	123 (0.38%)	**2.28 [1.91; 2.73]**	130 (0.09%)	70 (0.20%)	**2.15 [1.70; 2.72]**	57 (0.06%)	40 (0.11%)	**1.94 [1.42; 2.64]**
**SBP (mmHg)** F	116 (12.4)	118 (13.0)	**1.02 [1.01; 1.02]**	124 (15.0)	130 (17.1)	**1.03 [1.02; 1.03]**	131 (14.5)	134 (16.0)	**1.01 [1.01; 1.01]**	134 (14.5)	136 (15.9)	**1.01 [1.01; 1.01]**	136 (15.3)	138 (16.4)	**1.01 [1.00; 1.01]**

M	126 (12.7)	129 (17.3)	**1.02 [1.01; 1.03]**	129 (13.8)	134 (16.2)	**1.02 [1.02; 1.02]**	134 (14.5)	136 (15.9)	**1.01 [1.01; 1.01]**	135 (14.4)	137 (15.9)	**1.01 [1.01; 1.01]**	135 (15.1)	137 (15.7)	**1.00 [1.00; 1.01]**
**Low HDLc** F	6686 (26.5%)	61 (41.8%)	**1.99 [1.43; 2.76]**	53598 (25.8%)	2348 (36.8%)	**1.67 [1.59; 1.76]**	13561 (22.6%)	1502 (32.1%)	**1.60 [1.51; 1.70]**	21888 (23.4%)	4761 (31.9%)	**1.51 [1.46; 1.56]**	17999 (25.5%)	7978 (29.8%)	**1.28 [1.25; 1.31]**
M	6686 (26.5%)	61 (41.8%)	**1.35 [0.98; 1.87]**	29054 (25.0%)	2176 (33.4%)	**1.49 [1.41; 1.56]**	14588 (19.1%)	3070 (25.6%)	**1.43 [1.37; 1.49]**	10017 (16.5%)	3482 (20.9%)	**1.32 [1.27; 1.37]**	6027 (17.6%)	3325 (19.4%)	**1.18 [1.14; 1.23]**
**nonHDLc increase (10 mg/dl)** F	13.1 (3.52%)	13.7 (3.89%)	**1.05 [1.00; 1.09]**	15.4 (3.78%)	16.4 (4.09%)	**1.06 [1.06; 1.07]**	16.3 (3.51%)	16.2 (3.79%)	**0.99 [0.99; 1.00]**	15.9 (3.43%)	15.6 (3.59%)	**0.98 [0.97; 0.98]**	15.3 (3.49%)	15.1 (3.55%)	**0.98 [0.98; 0.98]**
M	15.0 (3.91%)	15.8 (5.09%)	**1.06 [1.02; 1.10]**	16.4 (3.87%)	16.9 (4.20%)	**1.03 [1.03; 1.04]**	16.0 (3.60%)	16.0 (3.90%)	**0.99 [0.99; 1.00]**	15.2 (3.41%)	15.0 (3.58%)	**0.98 [0.98; 0.98]**	14.4 (3.36%)	14.2 (3.47%)	**0.98 [0.97; 0.98]**
**LDLc> 190 mg/dl** F	412 (1.63%)	4 (2.74%)	**1.69 [0.63; 4.58]**	10697 (5.14%)	500 (7.84%)	**1.56 [1.42; 1.71]**	3580 (5.96%)	277 (5.92%)	**0.99 [0.88; 1.12]**	4060 (4.34%)	565 (3.79%)	**0.88 [0.80; 0.95]**	2308 (3.27%)	807 (3.02%)	**0.93 [0.86; 0.99]**
M	616 (3.48%)	9 (5.42%)	**1.59 [0.81; 3.11]**	6881 (5.93%)	511 (7.84%)	**1.34 [1.22; 1.46]**	3432 (4.49%)	636 (5.31%)	**1.17 [1.08; 1.27]**	1555 (2.56%)	450 (2.70%)	**1.04 [0.95; 1.15]**	556 (1.62%)	277 (1.61%)	**0.98 [0.87; 1.10]**
**SCORE2/OP**															
F, Low	11272 (100%)	81 (100%)	**Ref.**	105916 (89.6%)	3160 (71.5%)	**Ref.**	32883 (74.7%)	2197 (59.5%)	**Ref.**	60008 (79.4%)	8743 (69.1%)	**Ref.**	56040 (95.7%)	21413 (94.2%)	**Ref.**
M, Low	6139 (78.8%)	48 (69.6%)		30235 (44.3%)	1051 (23.8%)	**Ref.**	10398 (19.1%)	918 (10.1%)	**Ref.**	1152 (2.38%)	248 (1.78%)	**Ref.**	18 (0.06%)	5 (0.03%)	**Ref.**
F, Moderate	--	--	--	10857 (9.19%)	1019 (23.1%)	**3.07 [2.86; 3.30]**	9640 (21.9%)	1155 (31.3%)	**1.76 [1.64; 1.89]**	13968 (18.5%)	3399 (26.8%)	**1.61 [1.55; 1.68]**	2463 (4.20%)	1275 (5.61%)	**1.28 [1.21; 1.36]**
M, Moderate	1614 (20.7%)	15 (21.7%)	**1.19 [0.67; 2.12]**	34358 (50.3%)	2571 (58.3%)	**2.12 [1.97; 2.28]**	34817 (63.9%)	5304 (58.1%)	**1.69 [1.57; 1.81]**	29468 (60.9%)	6790 (48.8%)	**1.03 [0.91; 1.17]**	6527 (23.2%)	2525 (17.3%)	**1.24 [0.52; 2.98]**
F, High	--	--	--	1387 (1.17%)	239 (5.41%)	**5.52 [4.84; 6.29]**	1496 (3.40%)	343 (9.28%)	**3.25 [2.90; 3.64]**	1585 (2.10%)	518 (4.09%)	**2.12 [1.94; 2.32]**	81 (0.14%)	47 (0.21%)	**1.46 [1.10; 1.95]**

M, High	36 (0.46%)	6 (8.70%)	**20.1 [8.61; 47.0]**	3710 (5.43%)	789 (17.9%)	**5.73 [5.22; 6.28]**	9311 (17.1%)	2908 (31.9%)	**3.27 [3.04; 3.52]**	17786 (36.7%)	6879 (49.4%)	**1.65 [1.45; 1.87]**	21639 (76.8%)	12024 (82.6%)	**1.80 [0.75; 4.33]**
**Hypertension** F	2075 (0.77%)	47 (4.86%)	**6.56 [4.89; 8.79]**	90741 (8.77%)	5646 (29.4%)	**4.26 [4.13; 4.39]**	49539 (33.5%)	5090 (51.7%)	**2.07 [1.99; 2.16]**	99334 (48.1%)	18399 (64.4%)	**1.88 [1.83; 1.93]**	103655 (59.0%)	39635 (70.2%)	**1.54 [1.52; 1.57]**
M	3733 (1.29%)	92 (6.60%)	**5.37 [4.35; 6.63]**	70037 (7.75%)	6831 (23.1%)	**3.49 [3.40; 3.59]**	73173 (29.2%)	13353 (41.6%)	**1.67 [1.64; 1.71]**	65119 (44.0%)	19412 (55.3%)	**1.50 [1.47; 1.54]**	46096 (49.5%)	23024 (60.7%)	**1.48 [1.45; 1.51]**
**Triglycerides. > 150 mg/dl** F	3297 (12.1%)	28 (17.4%)	**1.52 [1.01; 2.29]**	40672 (18.9%)	2266 (33.0%)	**2.09 [1.99; 2.20]**	14638 (23.4%)	1569 (31.5%)	**1.49 [1.40; 1.58]**	24061 (24.5%)	4957 (31.5%)	**1.39 [1.34; 1.43]**	17945 (23.7%)	7832 (27.4%)	**1.19 [1.15; 1.22]**
M	6215 (31.2%)	74 (40.7%)	**1.51 [1.12; 2.03]**	53293 (41.1%)	3885 (51.9%)	**1.53 [1.46; 1.60]**	29419 (35.7%)	5458 (41.6%)	**1.26 [1.22; 1.30]**	17339 (26.8%)	5522 (31.0%)	**1.20 [1.16; 1.24]**	7225 (19.6%)	4099 (22.2%)	**1.11 [1.07; 1.14]**
**Atherogenic dyslipidemia** F	1226 (5.72%)	19 (14.7%)	**2.83 [1.74; 4.61]**	19568 (10.2%)	1237 (20.0%)	**2.17 [2.04; 2.31]**	6657 (11.4%)	814 (17.7%)	**1.65 [1.53; 1.78]**	10791 (11.7%)	2532 (17.2%)	**1.52 [1.46; 1.59]**	8097 (11.6%)	3826 (14.5%)	**1.27 [1.23; 1.32]**
M	2334 (13.9%)	27 (16.9%)	**1.26 [0.83; 1.90]**	17470 (15.4%)	1445 (22.4%)	**1.56 [1.47; 1.65]**	8384 (11.2%)	1824 (15.4%)	**1.41 [1.34; 1.48]**	4896 (8.23%)	1745 (10.6%)	**1.29 [1.23; 1.35]**	2257 (6.77%)	1369 (8.15%)	**1.18 [1.12; 1.25]**
**BMI > 30 kg/m2** F	8579 (21.0%)	65 (29.4%)	**1.57 [1.17; 2.09]**	47498 (31.2%)	2273 (48.5%)	**2.06 [1.94; 2.18]**	17257 (42.4%)	1967 (54.6%)	**1.60 [1.50; 1.71]**	31547 (43.3%)	6611 (53.3%)	**1.45 [1.40; 1.50]**	20431 (36.1%)	9546 (43.5%)	**1.28 [1.24; 1.31]**
M	3425 (19.7%)	38 (27.9%)	**1.57 [1.08; 2.29]**	28241 (31.4%)	2164 (42.4%)	**1.59 [1.50; 1.68]**	19470 (35.6%)	3672 (40.0%)	**1.18 [1.13; 1.23]**	15717 (32.6%)	5193 (36.9%)	**1.17 [1.13; 1.21]**	7592 (25.4%)	4529 (29.5%)	**1.13 [1.10; 1.17]**
**NAFLD** F	226 (0.08%)	2 (0.21%)	**2.47 [0.62; 9.88]**	4555 (0.44%)	265 (1.38%)	**3.12 [2.76; 3.52]**	1698 (1.15%)	187 (1.90%)	**1.63 [1.41; 1.88]**	2268 (1.10%)	461 (1.61%)	**1.44 [1.31; 1.58]**	934 (0.53%)	388 (0.69%)	**1.20 [1.09; 1.33]**
M	674 (0.23%)	7 (0.50%)	**2.15 [1.02; 4.52]**	6508 (0.72%)	384 (1.30%)	**1.80 [1.63; 2.00]**	3249 (1.30%)	576 (1.79%)	**1.36 [1.25; 1.48]**	1577 (1.06%)	462 (1.32%)	**1.21 [1.11; 1.33]**	477 (0.51%)	267 (0.70%)	**1.28 [1.14; 1.44]**
**eGFR ≤ 60 mL/min/1.73 m2**	91 (0.18%)	3 (1.11%)	**6.37 [2.04; 19.9]**	4100 (1.36%)	380 (4.61%)	**3.44 [3.11; 3.82]**	4004 (5.69%)	532 (9.57%)	**1.73 [1.58; 1.89]**	14600 (13.4%)	3455 (19.7%)	**1.57 [1.51; 1.63]**	31465 (36.1%)	14193 (43.5%)	**1.36 [1.33; 1.39]**

F															
M	94 (0.30%)	9 (3.63%)	**12.2 [6.27; 23.7]**	1551 (0.97%)	245 (2.91%)	**3.01 [2.65; 3.42]**	4027 (4.30%)	1035 (7.19%)	**1.68 [1.58; 1.79]**	9000 (12.1%)	3316 (16.5%)	**1.41 [1.36; 1.46]**	13734 (30.7%)	8012 (36.5%)	**1.31 [1.27; 1.34**
**UACR > 30 mg/gr** F	84 (4.79%)	2 (12.5%)	**2.84 [0.65; 12.5]**	1806 (6.35%)	270 (16.0%)	**2.72 [2.39; 3.10]**	735 (5.45%)	183 (12.6%)	**2.40 [2.06; 2.80]**	1574 (6.24%)	722 (13.9%)	**2.27 [2.10; 2.46]**	2281 (11.3%)	1635 (18.2%)	**1.79 [1.69; 1.89]**
M	106 (6.19%)	4 (16.0%)	**2.86 [0.98; 8.34]**	1623 (7.96%)	394 (21.6%)	**3.03 [2.71; 3.39]**	1964 (9.36%)	828 (19.7%)	**2.24 [2.07; 2.41]**	1966 (10.4%)	1175 (19.1%)	**1.92 [1.80; 2.04]**	1844 (17.1%)	1484 (23.9%)	**1.59 [1.50; 1.69]**
**High risk alcohol consumption** F	333 (1.20%)	0 (0.00%)	**0.00 [0.00;.]**	2176 (1.38%)	95 (2.03%)	**1.44 [1.18; 1.77]**	432 (1.03%)	41 (1.18%)	**1.09 [0.80; 1.49]**	481 (0.67%)	76 (0.65%)	**0.93 [0.74; 1.16]**	269 (0.48%)	89 (0.42%)	**0.90 [0.73; 1.11]**
M	985 (4.24%)	9 (4.89%)	**1.09 [0.55; 2.16]**	6614 (6.32%)	504 (9.45%)	**1.53 [1.39; 1.68]**	3621 (6.50%)	764 (8.58%)	**1.24 [1.15; 1.34]**	2141 (4.45%)	672 (5.04%)	**1.08 [1.00; 1.17]**	645 (2.19%)	370 (2.54%)	**1.09 [0.98; 1.21]**
**DI (U5)** F	35096 (18.6%)	152 (21.2%)	**1.58 [1.23; 2.03]**	127346 (16.9%)	2973 (21.0%)	**1.58 [1.49; 1.66]**	18034 (16.2%)	1424 (19.2%)	**1.48 [1.38; 1.60]**	26129 (17.1%)	4121 (20.1%)	**1.40 [1.34; 1.46]**	16820 (14.5%)	5517 (16.1%)	**1.22 [1.18; 1.26]**
M	45308 (22.4%)	254 (25.5%)	**1.47 [1.19; 1.81]**	129630 (20.2%)	4921 (23.1%)	**1.32 [1.27; 1.38]**	29595 (16.6%)	4308 (18.6%)	**1.30 [1.25; 1.36]**	18040 (17.2%)	4609 (18.8%)	**1.20 [1.15; 1.24]**	9207 (15.6%)	3809 (16.7%)	**1.17 [1.13; 1.22]**

Data are number (percentage) or mean (standard deviation) as indicate. To convert cholesterol to mmol/L, multiply values by 0.0259. To convert triglycerides to mmol/L multiply values by 0.0113.

Low HDLc: <50mg/dl (women) or <40mg/dl (men)

F: female, M: male, T2DM: Type 2 diabetes, T1DM: Type 1 diabetes, SBP: systolic blood pressure, BMI: Body Mass Index, NAFLD: non-alcoholic fatty liver disease, eGFR; glomerular filtration rate, UACR: urinary albumin-to-creatinine ratio, DI: Deprivation Index

Missing data were frequent(see supplementary information): smoking (41%), systolic blood pressure (69%), total cholesterol (69%), HDL cholesterol (77%), non-HDL cholesterol (77%), SCORE2/OP (84%), triglycerides (75%), atherogenic dyslipidemia (78%), body mass index, BMI (kg/m2) (82%), eGFR (CKD-EPI) mL/min/1.73m2 (69%), urinary albumin-to-creatinine ratio (UACR) (94%), alcohol consumption (81%), deprivation index (DI) (29%)

Table S6. Mortality during follow-up by age groups and sex

Age groups	All (n=3769563)		Women (n=1949847)		Men (n=1819716)	
	**Survived**	**Death**	**Survived**	**Death**	**Survived**	**Death**
All	3581300 (95.01)	**188263 (4.99)**	1860333 (95.41)	**89514 (4.59)**	1720967 (94.57)	**98749 (5.43)**
Young (<35 y)	560270 (99.72)	1577 (0.28)	271093 (99.80)	546 (0.20)	289177 (99.64)	1031 (0.36)
Early adulthood (35 – 55/60 y)	1961003 (98.73)	25284 (1.27)	1042671 (98.98)	10793 (1.02)	918332 (98.45)	14491 (1.55)
Middle adulthood (55/60 – 65 y)	419653 (95.39)	20278 (4.61)	153251 (97.24)	4350 (2.76)	266402 (94.36)	15928 (5.64)
Young old (65 – 75 y)	381114 (91.15)	36991 (8.85)	220577 (93.91)	14302 (6.09)	160537 (87.62)	22689 (12.38)
Middle-to-very old (> 75 y)	259260 (71.34)	104133 (28.66)	172741 (74.37)	59523 (25.63)	86519 (65.98)	44610 (34.02)

Data are number (and percentage). Y**oung**, < 35 y; **early adulthood**, 35-55/60 y (men/women); **middle adulthood**, 55/60-65 (men/women); **young old**,65-75 y; and **middle-to-very old**, > 75 years.

Table S7. Prevalence of first cardiovascular event before date of death

	All			Women			Men		
	All death	Free-CV event	CV-event	All death	Free-CV event	CV-event	All death	Free-CV event	CV-event
All	N=188263	N=131436 (69.82)	N=56827 (30.18)	N=89514	N=62033 (69.30)	N=27481 (30.70)	N=98749	N=69403 (70.28)	N=29346 (29.72)
Young (<35 y)	1577	1433 (90.87)	144 (9.13)	546	481 (88.10)	65 (11.90)	1031	952 (92.34)	79 (7.66)
Early adulthood (35 – 55/60 y)	25284	21550 (85.23)	3734 (14.77)	10793	9268 (85.87)	1525 (14.13)	14491	12282 (84.76)	2209 (15.24)
Middle adulthood (55/60 – 65 y)	20278	15626 (77.06)	4652 (22.94)	4350	3508 (80.64)	842 (19.36)	15928	12118 (76.08)	3810 (23.92)
Young old (65 – 75 y)	36991	25956 (70.17)	11035 (29.83)	14302	10236 (71.57)	4066 (28.43)	22689	15720 (69.28)	6969 (30.72)
Middle-to-very old (> 75 y)	104133	66871 (64.22)	37262 (35.78)	59523	38540 (64.75)	20983 (35.25)	44610	28331 (63.51)	16279 (36.49)

Data are number and percentage. **Young**, < 35 y; **early adulthood**, 35-55/60 y (men/women); **middle adulthood**, 55/60-65 (men/women); **young old**,65-75 y; and **middle-to-very old**, > 75 years
