# Supplementary figures and images for: First manifestation of cardiovascular disease according to age and sex in a Mediterranean country
Source: Front Cardiovasc Med. 2024 Sep 17;11:1403363. doi: 10.3389/fcvm.2024.1403363 (PMC11443696; doi:10.3389/fcvm.2024.1403363)

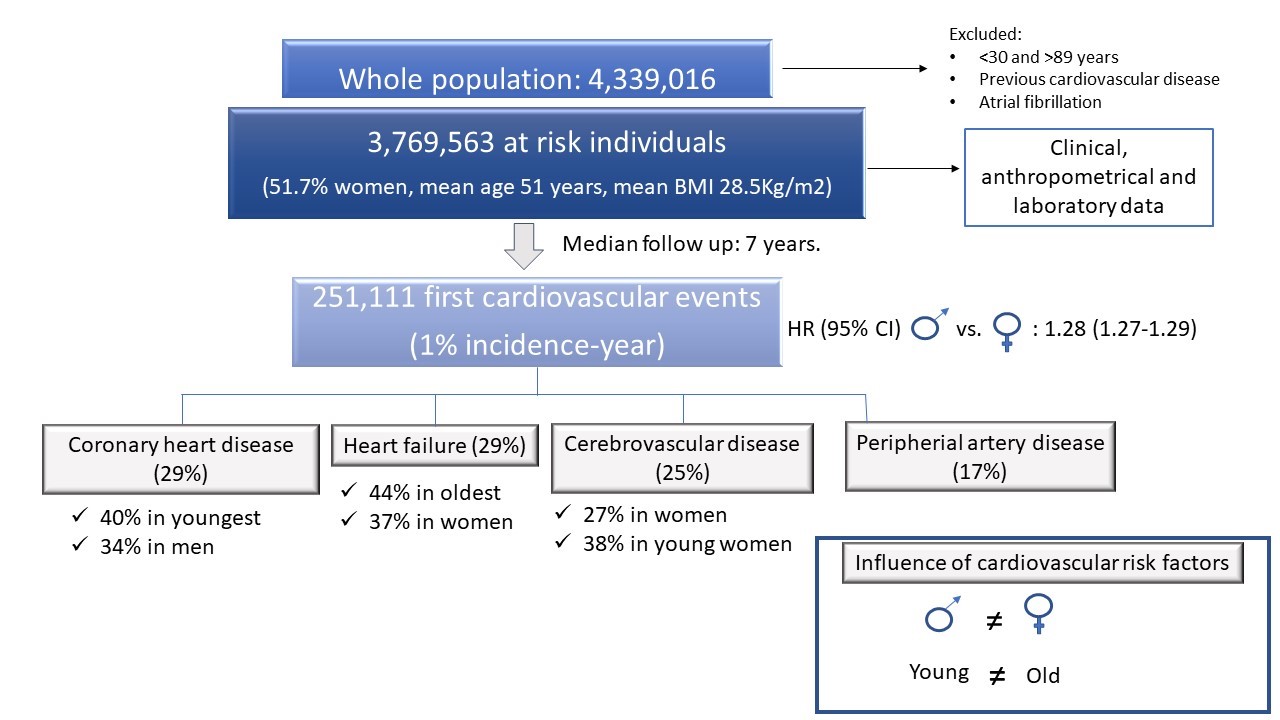

Supplement: Supplementary file 2 [file Image2.jpeg]
